# Real-world nudging, pricing, and mobile physical activity coaching was insufficient to improve lifestyle behaviours and cardiometabolic health: the Supreme Nudge parallel cluster-randomised controlled supermarket trial

**DOI:** 10.1186/s12916-024-03268-4

**Published:** 2024-02-02

**Authors:** Josine M. Stuber, Joreintje D. Mackenbach, Gert-Jan de Bruijn, Marleen Gillebaart, Jody C. Hoenink, Cédric N. H. Middel, Denise T. D. de Ridder, Yvonne T. van der Schouw, Edith G. Smit, Elizabeth Velema, Anne L. Vos, Wilma E. Waterlander, Jeroen Lakerveld, Joline W. J. Beulens, on behalf of the SUPREME NUDGE consortium, Femke Rutters, Stephanie Blom, Femke E. de Boer, Michel C.A. Klein, Jacqueline E. W. Broerse, Tjerk-Jan Schuitmaker-Warnaar, Ivonne Sluijs, Marjolein C. Harbers

**Affiliations:** 1 Epidemiology and Data Science, Amsterdam UMC Location Vrije Universiteit Amsterdam, De Boelelaan 1117, Amsterdam, the Netherlands; 2 Amsterdam Public Health, Amsterdam, the Netherlands; 3 Upstream Team, www.upstreamteam.nl, Amsterdam UMC, De Boelelaan 1117, Amsterdam, the Netherlands; 4 Department of Communication Science, University of Antwerp, St-Jacobstraat 2, 2000 Antwerp, Belgium; 5 Department of Social, Health and Organizational Psychology, Utrecht University, Utrecht, the Netherlands; 6 Centre for Diet and Activity Research, MRC Epidemiology Unit, School of Clinical Medicine, Institute of Metabolic Science, University of Cambridge, Cambridge, CB2 0QQ UK; 7 Julius Center for Health Sciences and Primary Care, University Medical Center Utrecht, Utrecht University, Universiteitsweg 100, Utrecht, the Netherlands; 8 Amsterdam School of Communication Research, University of Amsterdam, Nieuwe Achtergracht 166, Amsterdam, the Netherlands; 9 Netherlands Nutrition Centre (Voedingscentrum), Bezuidenhoutseweg 105, The Hague, The Netherlands; 10 Public and Occupational Health, Amsterdam UMC Location University of Amsterdam, Meibergdreef 9, Amsterdam, the Netherlands

**Keywords:** Food environment, Grocery store, Choice architecture, Prevention, Obesity, Cardiovascular disease

## Abstract

**Background:**

Context-specific interventions may contribute to sustained behaviour change and improved health outcomes. We evaluated the real-world effects of supermarket nudging and pricing strategies and mobile physical activity coaching on diet quality, food-purchasing behaviour, walking behaviour, and cardiometabolic risk markers.

**Methods:**

This parallel cluster-randomised controlled trial included supermarkets in socially disadvantaged neighbourhoods across the Netherlands with regular shoppers aged 30–80 years. Supermarkets were randomised to receive co-created nudging and pricing strategies promoting healthier purchasing (*N* = 6) or not (*N* = 6). Nudges targeted 9% of supermarket products and pricing strategies 3%. Subsequently, participants were individually randomised to a control (step counter app) or intervention arm (step counter and mobile coaching app) to promote walking. The primary outcome was the average change in diet quality (low (0) to high (150)) over all follow-up time points measured with a validated 40-item food frequency questionnaire at baseline and 3, 6, and 12 months. Secondary outcomes included healthier food purchasing (loyalty card-derived), daily step count (step counter app), cardiometabolic risk markers (lipid profile and HbA1c via finger prick, and waist circumference via measuring tape), and supermarket customer satisfaction (questionnaire-based: very unsatisfied (1) to very satisfied (7)), evaluated using linear mixed-models. Healthy supermarket sales (an exploratory outcome) were analysed via controlled interrupted time series analyses.

**Results:**

Of 361 participants (162 intervention, 199 control), 73% were female, the average age was 58 (SD 11) years, and 42% were highly educated. Compared to the control arm, the intervention arm showed no statistically significant average changes over time in diet quality (*β ﻿−* 1.1 (95% CI ﻿− 3.8 to 1.7)), percentage healthy purchasing (*β* 0.7 ( ﻿− 2.7 to 4.0)), step count (*β ﻿−* 124.0 (﻿− 723.1 to 475.1), or any of the cardiometabolic risk markers. Participants in the intervention arm scored 0.3 points (0.1 to 0.5) higher on customer satisfaction on average over time. Supermarket-level sales were unaffected (*β* − 0.0 (− 0.0 to 0.0)).

**Conclusions:**

Co-created nudging and pricing strategies that predominantly targeted healthy products via nudges were unable to increase healthier food purchases and intake nor improve cardiometabolic health. The mobile coaching intervention did not affect step count. Governmental policy measures are needed to ensure more impactful supermarket modifications that promote healthier purchases.

**Trial registration:**

Dutch Trial Register ID NL7064, 30 May 2018, https://www.onderzoekmetmensen.nl/en/trial/20990

**Supplementary Information:**

The online version contains supplementary material available at 10.1186/s12916-024-03268-4.

## Background

Cardiometabolic diseases such as cardiovascular diseases and type 2 diabetes are leading causes of morbidity and mortality worldwide [1, 2]. Unhealthy diets and physical inactivity are key modifiable risk factors, which are strongly socioeconomically patterned [3–5]. Individual-level interventions to improve lifestyle behaviours such as behavioural counselling are generally not sustainable beyond their duration [6, 7]. Such interventions rely heavily on individual resources like knowledge and motivation and inadequately reach vulnerable populations including those with a low socioeconomic position (SEP) [8–10]. Interventions that modify the environmental context surrounding lifestyle choices can enhance healthier lifestyle behaviours for all individuals exposed to this context and reduce cardiometabolic risk on a population level [11].

The supermarket is an important context to modulate food choices and is where about 80% of foods are purchased in the Netherlands [12]. Yet, only 21% of products sold in Dutch supermarkets are recommended by the Dutch dietary guidelines [13]. Nudging and pricing strategies may help customers choose more healthy products. Nudging—environmental changes that promote a certain choice, without removing the alternative choice—has been shown to increase healthier purchasing or sales, mainly in highly controlled experimental contexts [14], while mixed effects are observed in real-world contexts [15–18]. Pricing strategies such as food taxes and subsidies are effective in stimulating healthier choices [19, 20] and are a recommended policy of the World Health Organization [21]. However, impactful food pricing policies are politically difficult to implement and therefore potentially not sufficient to achieve a large-scale impact on health. Implementing various types of nudging and pricing strategies simultaneously across food groups are probably needed to substantially improve dietary patterns [18, 22].

Wearable devices or smartphones can provide context-specific cues to stimulate physical activity by prompting at decision-making moments [23, 24], rendering them context-specific, yet not limited to a physical setting or place. The promotion of walking is a relevant target for preventing cardiometabolic diseases for individuals across SEP and age groups; it is free of cost to users and easily incorporated into daily activities. Existing mobile coaching strategies generally focus on the motivation to change physical activity and rarely consider the individual context [25–28]. Providing context-specific coaching messages may enhance the context action relationship and promote walking habits [29].

Most previous real-world studies evaluated effects of nudging, pricing, and mobile coaching strategies separately, with mixed or small effects [15, 18, 20, 30–32]. Only two real-world studies evaluated effects of a combined nudging and pricing intervention, but these targeted only a single or combination of specific food groups and evaluated short-term effects (≤ 6 months) on supermarket-level sales [33, 34]. Information on long-term and real-world effects of nudging, pricing, and mobile coaching strategies on various lifestyle behaviours and health outcomes would provide a strong methodological underpinning on their effectiveness across populations [35].

Within the Supreme Nudge project [36], we evaluated the real-world effects of nudging and pricing strategies promoting healthy products and a mobile coaching app promoting walking on individual-level diet quality, purchasing behaviour, walking behaviour, cardiometabolic risk markers, customer satisfaction, and various psychosocial factors among Dutch adults after 6 to 12 months compared to no interventions. We hypothesised that nudging, pricing, and mobile physical activity coaching would improve healthier food purchasing and intake, daily step count, customer satisfaction, and cardiometabolic risk markers. Supermarket-level sales data were used to assess whether selective trial participation influenced our results, by exploring changes in supermarket sales trends in intervention supermarkets compared to control supermarkets.

## Methods

### Trial design

We conducted a parallel cluster-randomised controlled trial (RCT)—the Supreme Nudge trial—in 12 Dutch supermarkets from one chain in socially disadvantaged neighbourhoods. Supermarkets were randomised to a control arm (*n* = 6) or intervention arm receiving nudging and pricing strategies to promote healthy food and beverage purchases (*n* = 6). Participants were additionally randomised to a mobile physical activity coaching app intervention (‘SNapp’), receiving either a mobile coaching app and a step counter app (intervention arm) or only the step counter app (control arm). The duration of intervention implementation was six (*n* = 4 supermarket clusters) to 12 months (*n* = 8) depending on supermarket enrolment date.

More detailed information on the trial design is published in a protocol paper [37], including a correction paper detailing protocol changes due to the COVID-19 pandemic [38]. We also briefly summarise these protocol changes further down. The current paper focusses mainly on the supermarket-related outcomes. In a separate paper, we will describe and evaluate the mobile physical activity coaching app intervention in more detail. This trial was prospectively registered in the Dutch Trial Register (ID NL7064) on 30 May 2018. The reporting this paper presents is in accordance with the 2010 Consort statement for cluster-randomised trials [39].

### Supermarkets

Eligible supermarkets were regular stores (i.e. no convenience store) located in socially disadvantaged neighbourhoods (below average postal code SEP-scores according to The Netherlands Institute for Social Research) [40] and had implemented an adapted cash-register system to allow for pricing strategies. Of 69 eligible supermarkets, we selected 12 balancing the lowest number of competing supermarkets within a 2-km radius and the highest number of potentially eligible residents living in the neighbourhood. There were no systematic differences between supermarkets at baseline.

### Participants

Eligible individuals were aged 30–80 years, regular shoppers at a participating supermarket (self-reported to purchase > 50% weekly groceries at a participating supermarket), planning on continuing shopping there for the study period, and could communicate in Dutch. Participants had to provide written informed constant prior to study enrolment. For additional individual-level randomisation to the mobile coaching app, participants had to use a smartphone with a mobile data plan and at least Android 8 or iOS 13, have experience with mobile text messaging, and be able to climb a flight of stairs.

### Recruitment

We used a mix of passive and active recruitment strategies prior to the intervention implementation, as described elsewhere [41]. Passive strategies entailed, for example, online news articles in local newspapers, flyers and invitation letters sent to every household in the supermarkets’ neighbourhood, flyers in supermarkets, and e-mail invitations to supermarket customer panels. Active recruitment strategies included word-of-mouth promotion among included participants and in-store recruitment by research staff.

### Intervention

#### Nudging and pricing strategies

Supermarket interventions consisted of a combination of nudging and pricing strategies to promote healthier food purchasing. We targeted a wide range of healthy product groups (i.e. vegetables, fruits, whole-grain products, fish, low-fat milk and yogurt, low-fat cheese, legumes, unsaturated fats, unsalted nuts, sugar-free bottled beverages, and tea) to promote a healthy dietary pattern which is in accordance with the Dutch dietary guidelines [42]. Strategies were developed in a co-creative process with supermarket stakeholders and interventionists [37, 43]. Supermarket interventions were implemented by supermarket staff so that they would be sustainable and scalable beyond the duration of the project. We used data of the Netherlands Nutrition Centre to classify products recommended (‘healthy’) or non-recommended (‘unhealthy’) [44]. Each of the included supermarkets offered around 12,500 foods and beverages, of which 19% were classified as healthy (Additional file 1: Table S1).

We used the Typology of Interventions in Proximal Physical Micro-Environments (TIPPME) to classify nudges into placement nudges (availability and the positioning of products) and property nudges (functionality or design of products, or highlighting specific product information) [45]. Our placement nudges adjusted the shelf location and increased the number of healthy products prominently placed. The property nudges consisted of information symbols highlighting healthy product’s *tastiness*, *convenience*, or *popularity*, applied as shelf feedback strips, shelf-stoppers, healthy suggestions shelf-banners, and shelf-labels. The self-labels targeted all available healthy products within a food group (Table 1). Nudges were implemented for the entire trial period on approximately 1100 healthy products simultaneously (i.e. 9% of supermarket assortment). Photographic examples can be found in Additional file 2: Figures S1a–k.

**Table 1:** Overview of implemented supermarket interventions in the Supreme Nudge trial

Intervention strategies	Components	Explanation of intervention	Targeted product groups	Static or dynamic
Placement nudges	Healthy shelf layout	Healthy products placed at eye level	Shelves including pasta and rice products, bread substitutes, and breakfast cereals	Static
	Healthy checkout	One check-out till presenting only healthy products	Sugar-free beverages, nuts, snack vegetables, dried fruits, whole-grain crackers, instead of confectionery products	Static
	Healthy end of aisle	Promoting only healthy products on a prominent end of aisle; monthly product switch	Whole-grain products, oils, canned fish/legumes/ tomatoes, nuts, natural peanut butter, sugar-free beverages	Dynamic
	Healthy aisle baskets	Three to four aisle baskets filled with healthy products; monthly product switch	Nuts, canned fish/legumes/tomatoes, whole-grain products	Dynamic
Property nudges	Shelf-labels	Symbols which highlighted the product’s *tastiness* (smiley symbol), *convenience* (stopwatch symbol indicating ease of preparation) or *popularity* (thumbs-up symbol)	All healthy products across a range of food groups (i.e. vegetables, fruits, all whole-grain products, fish, milk and yogurt, cheese, legumes, butters and oils, nuts, sugar-free bottled beverages, and tea)	Static
	Shelf feedback strips	Positive feedback strip with popularity symbol	Underneath whole-grain bread, fresh fish and snack vegetables	Static
	Shelf-stoppers	Shelf cards highlight one healthy product per product group; monthly product switch	At each shelf including the shelf-labels	Dynamic
	Healthy suggestions shelf-banners	Seasonal shelf-banners suggesting different healthy product combinations using the convenience symbol	Whole-grains, legumes and bread substitutes	Static
	Explanation of nudging symbols	The symbols are introduced on the shopping cart handles, and also shown on shopping cart/baskets boards and the checkout divider bars	N/A	Static
Pricing strategies	Price reductions	Price reductions were − 25% or − 10% when combined with price increases in the same food group; three-weekly product switch	Healthy product groups: vegetables, fruit, whole-grain products, fresh fish, low and medium-fat dairy, low-salt legumes, butters and oils with unsaturated fats, unsalted nuts, sugar-free beverages	Dynamic
	Price increases	Price increases were + 15%; three-weekly product switch	Unhealthy product groups: non-whole-grain products, processed/salted fish, high fat and sugary dairy, high-salt legumes, butters and oils with saturated fats, salted nuts, sugary beverages	Dynamic

*Static* Intervention targeting the same food group(s) for the complete trial period, *Dynamic* Interventions altered over targeted products or food group(s) during the trial period. *N/A* Not applicable

Pricing strategies consisted of salient price reductions of healthy products and non-salient price increases of unhealthy products, wherever possible, within the same food group (Table 1). Price reductions were by 25% in food groups with only healthy options (e.g. fresh vegetables) or by 10% on healthy products when combined with a 15% increase on unhealthy products in the same food group. The supermarket chain allowed a maximum of 200 price changes per week and products in regular weekly price promotions were excluded from the pricing strategies. Pricing strategies targeted 3% of the total assortment and were implemented for ~ 3 consecutive weeks in one or multiple food groups, after which they switched to other food groups (Additional file 1: Table S2).

#### Mobile physical activity coaching app

The mobile physical activity coaching app consisted of a smartphone step counter app and dynamically tailored coaching content [46]. The app continuously quantified step count through the smartphone’s built-in pedometer or accelerometer. GPS data comparing participants’ location to a database of pre-selected locations suitable for walking every 2 min served as input for the coaching content. Three types of content were delivered via push notifications in a Telegram Messenger chat account: (1) feedback tailored to participants’ step count levels, (2) contextually tailored prompts when participants were near walking locations, and (3) advice tailored to behavioural change technique preferences measured at baseline. Coaching content was adapted to be readily understood by individuals with a low SEP (language level ‘Dutch B1’). A server-based Python program that interacted with databases containing user data and coaching messages automatically selected appropriate messages to send to participants using a rule-based system. Participants received at least two and maximally six messages daily.

### Study outcomes and data collection

Measurements took place at baseline (T0), after 3 months (T1), after 6 months (T2), and after 12 months (T3). The primary outcome was the change in total diet quality (Dutch Healthy Diet 2015 index) from baseline until 12 months [47], measured with a validated short 40-item food frequency questionnaire (FFQ). The short FFQ asked about the average dietary intake during the past month from 15 components representing the food-based Dutch dietary guidelines of 2015. For each component, a score between 0 and 10 was assigned, resulting in a total score ranging from 0 (low diet quality) to 150 (high diet quality) [48, 49]. Secondary outcomes were as follows: the total percentage of healthier food purchasing (calculated based on grams purchased derived from weekly data collected via loyalty cards), daily step counts (step counter app) [46], cardiometabolic risk markers self-assessed by participants at home (glycated haemoglobin (HbA1c), low-density lipoprotein (LDL) cholesterol, high-density lipoprotein (HDL) cholesterol, total cholesterol (TC), TC/HDL-ratio, triglycerides (TG) collected via a finger prick measurement, and waist circumference via a measuring tape, presented and analysed stratified by sex) [37], and questionnaire items relating to overall customer satisfaction, food-decision styles in relation to vegetables and to snacks (reflective, habitual, and impulsive) [50–54], social cognitive factors related to nudges (heath goals, healthy shopping, perceived social norm, and attractiveness of healthy foods) [55–60], and acceptance and awareness of nudges after six or 12 months. The purchase data were collected weekly over the complete duration of the intervention and recalculated to an average purchase per blocks of 4 weeks, leading to 13 time points. Step count data was collected daily (Additional file 1: Table S3 [37, 38]). As explorative outcomes, we described medication use, diet quality scores of various food groups, and percentage of healthy purchasing within various food groups per time point. Further details on data collection methods are described elsewhere [37, 41].

At the supermarket-level, we explored whether nudging and pricing strategies changed supermarket-level sales trends to account for potential selective trial participation [61]. We used sales data of the intervention period plus 6 months of pre-intervention data. Sales data comprised the number of products and Euros spent on healthy and unhealthy products per supermarket per week, based on 63 product groups which were pre-defined by the supermarket chain. We primarily investigated changes in the total percentage of healthy product sales of all sales in intervention supermarkets compared to control supermarkets during the intervention period, while taking into account the pre-intervention sales trends. We assigned the 63 product groups into 10 overarching food groups and examined the percentage change in sales from: fruits, vegetables, legumes, and nuts, grain products, milk and yogurt products, cheese, meat products, meat substitutes and eggs, fish, oils, fats and herbs and spices, non-alcoholic beverages, products from all remaining food products (e.g. pre-packaged meals, and baking products), and sweet and savoury snacks of total sales. Lastly, we examined changes in total sales revenue (Euros).

We monitored the implementation fidelity of the nudging and pricing strategies monthly to bi-monthly as the trial progressed. Research staff visited intervention supermarkets and quantitatively scored intervention components separately by their implementation status (none/minimal implementation scored 1, to (almost) optimal implementation scored 5). A summary score of all components was calculated for each supermarket by time point.

### Sample size

The trial required 141 participants in each supermarket arm to detect an average change of 5 points in the diet quality score by the end of the intervention period, assuming a 14.1 point standard deviation (SD) of the average change (power = 80%; two-sided alpha = 0.05). The expected 5-point change was based on results from a virtual supermarket experiment which indicated a 4% increase in healthier purchasing following a combination of nudging and salient pricing strategies [22]. We did not use a design factor since baseline data of diet quality among participants from the first eight enrolled supermarkets showed no correlation for observations between study sites (intra-cluster correlation coefficient (ICC) = 0.00) [37]. To allow for 25% drop out, 176 participants needed to be recruited per arm, resulting in 352 participants.

### Randomisation and blinding

Twelve supermarkets were block-randomised by the first author to the control arm or the intervention arm, using a web-based random-number generator tool with blocks of four supermarkets. Individual-level randomisation in the mobile physical activity coaching app intervention or only the step counter app was conducted via a cloud-based data-management software application (Castor Electronic Data Capture). We used variable block randomisation alternating block sizes of two, four, and six, with equal weight for both groups. A research assistant conducted the randomisation of participants eligible for the coaching app intervention.

Blinding of participants was not feasible due to the nature of the interventions. Participants were not actively informed about the content of the supermarket interventions and not about the intervention allocation of their supermarket location, not prior to their study enrolment nor during the trial. Those randomised in the coaching app intervention were actively informed since they were requested to install the app(s).

### Brief summary of protocol changes

Due to the COVID-19 pandemic and its associated governmental restrictions (e.g. social distancing measures), the main protocol underwent some notable changes [38]. As we were unable to perform physical measurements face-to-face, we abandoned planned blood pressure measurements. Instead, waist circumference and blood markers including HbA1c and lipid profile were measured remotely via a self-assessed finger prick. Furthermore, the lack of active recruitment opportunities required a revision of recruitment goals. Due to a lower expected response rate, we recruited from twelve rather than eight supermarket locations. Due to the fixed project end-date, the additional four stores could only be followed-up for six instead of 12 months. The expected lower response rates also forced us to combine intervention arms, leading us to comparing a combined nudging and pricing arm to a control arm rather than also investigating the independent effect of nudging strategies. Finally, in response to the expected lower response rates and participant burden of self-assessment of cardiometabolic risk markers, we revised our primary outcome to only include changes in diet quality, as was already part of the primary outcome, and to exclude systolic blood pressure, LDL cholesterol, and HbA1c from the primary outcome. These changes required us to recruit 352 participants instead of the originally planned 1485.

### Statistical analyses

Population characteristics described were stratified by trial arm to visually inspect potential baseline differences as mean (SD) for normally distributed continuous variables, median (IQR) for skewed continuous variables, or frequencies (percentage) for categorical variables.

For evaluation of the primary outcome, we used a linear mixed model with group allocation as independent variable and the diet quality score at T1, T2, and T3 as dependent variable. The model included the covariates diet quality at T0, to account for regression to the mean, and time as categorical variable, considering the varying number of missing values per time point. Interaction between time and treatment indicated no time-specific intervention effects so this interaction was left out of the model. All models included random intercepts at the participant-level and the supermarket-level. The addition of random slopes did not improve the model fit and were therefore not included. Residuals were confirmed to be normally distributed via residual plots. Missing data were not imputed since mixed models use the maximum available data, even if participants had incomplete data. We reported the average within- and between group change in diet quality over all follow-up time points with a 95% confidence interval (CI) for the intervention arm, compared to the control arm and compared to the baseline measurement. We also reported the effect sizes on total diet quality at T1 and T2.

Changes in all secondary outcomes with the exception of step counts were analysed as described above for the primary outcome. Analysis of step count data followed a similar approach, but with only a random intercept at the participant-level, since their appeared no clustering of step count data between study sites, and including adjustment for smartphone operating system and sensor type used for step count tracking. Variables related to acceptance of nudges were only measured at T3 and therefore presented descriptively per arm by their mean (SD) or frequency (%).

Participants were analysed according to their initial treatment assignment (intention-to-treat), and we included participants who had a baseline measurement and at least one follow-up measurement of diet quality; this was considered our analytical sample. For secondary outcomes, we used an analytical sample per outcome, including those participants with at least a baseline measurement and one follow-up measurement for that outcome, leading to varying sample sizes by outcome.

We conducted several sensitivity analyses. To assess the influence of changes in medication on the cardiometabolic risk markers, we explored medication use over time between the trial arms with descriptive statistics. Descriptive statistics were presented for diet quality by food group and the percentage of healthier food purchasing by food group over time, to provide insights for each time point. We assessed effect modification of nudging and pricing strategies on diet quality by sex, age, and educational level by adding an interaction term between the trial arm and the potential effect modifier to the primary outcome model. When the interaction term was statistically significant (*p* < 0.10), these effect modifiers were used to explore interaction effects on relevant secondary outcomes. Significant interaction effects were plotted to visualise their direction.

For each explorative supermarket-level outcome, we described the average (mean (SD)) pre-intervention and post-intervention sales for both the intervention and the control supermarkets. We conducted controlled interrupted time series analyses based on a linear mixed model [62, 63]. For each outcome, we tested for changes in the average sales trends over time in intervention supermarkets compared to the control supermarkets, treating the pre-intervention period sales trends as fixed effects and including a random intercept for supermarkets. All analyses excluded the first week of the intervention implementation to allow time for the intervention to be implemented. We modelled changes in sales outcomes as an immediate step change, which assumed an immediate and stable effect of the intervention. Moreover, the average implementation fidelity scores per supermarket per time point were graphically presented, and the median score (range) over time is presented for all intervention stores combined. As a sensitivity analysis, the intervention supermarket with the lowest implementation fidelity score over time was excluded from the analyses of the total percentage of healthy product sales of all sales.

We did not account for multiple testing since our primary, secondary, and explorative analyses all reflect different outcome constructs and were planned in our study protocol. We used a two-sided alpha of 0.05 to define statistical significance, with the exception of the interaction tests for which we used an alpha of 0.10. Analyses were conducted using R statistical software (version 4.2.1), except for the step count data (SPSS version 27).

## Results

### Individual-level results

**Fig. 1:**
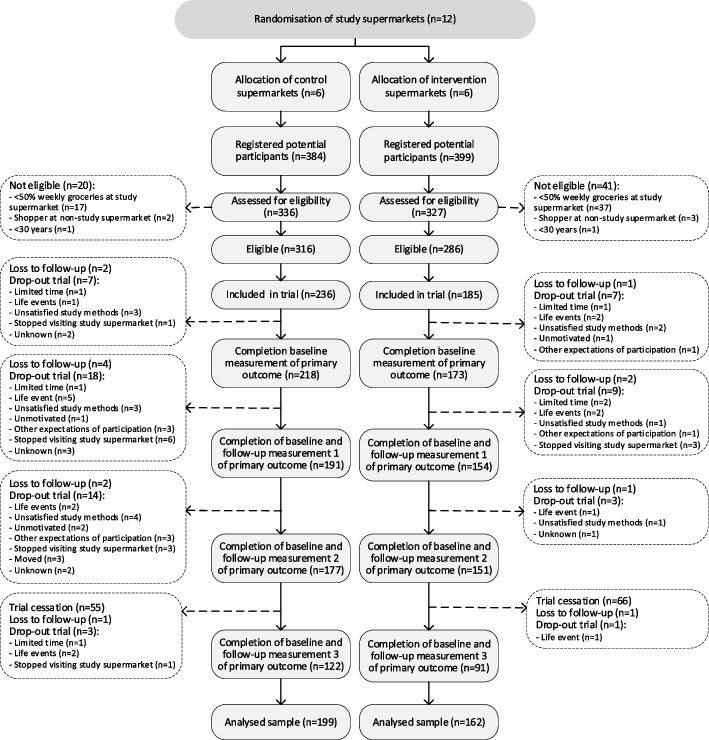
Participant flowchart of the Supreme Nudge trial

Recruitment ran from mid-January 2021 to November 2021. In total, 783 individuals registered for participation, of whom 602 were eligible and 421 signed informed consent (Fig. 1). Overall, 199 participants in the control arm and 162 in the intervention arm completed the baseline measurement and at least one follow-up measurement of the primary outcome, thereby reaching our target sample size of 360 participants [37]. Across trial arms, 73% of participants were female, 42% were highly educated and the average age was 58 (SD 11) years. The population characteristics were equally balanced between arms (Table 2) and across clusters [41], with the potential exception of prevalence of hyperlipidaemia.

**Table 2:** Population characteristics and baseline measurements in the Supreme Nudge trial (*n* = 361)

	Control supermarkets (*n* = 199)	Intervention supermarkets (*n* = 162)
*Baseline characteristics*		
Age, years, mean (SD)	57.2 (10.2)	58.9 (11.5)
Female^a^, *n* (%)	142 (71.4)	120 (74.1)
*Educational attainment, n (%)*		
Low	38 (19.1)	46 (28.4)
Medium	76 (38.2)	50 (30.9)
High	85 (42.7)	66 (40.7)
Household size, *n* adults^a^ (median [IQR])	2.0 [1.0]	2.0 [1.0]
Household size, *n* children^a^, (median [IQR])	0.0 [1.0]	0.0 [1.0]
*Smoking status*^a^*, n (%)*		
Current smoker	9 (4.5)	11 (6.8)
Irregular smoker	4 (2.0)	3 (1.9)
Former smoker	95 (47.7)	74 (45.7)
Never smoked	91 (45.7)	73 (45.1)
Prevalent type 2 diabetes^a^, *n* (%)	15 (7.5)	8 (4.9)
Medication for type 2 diabetes^a^, *n* (%)	14 (7.0)	8 (4.9)
Prevalent hypertension^a^, *n* (%)	32 (16.1)	26 (16.0)
Medication for hypertension^a^, *n* (%)	37 (18.6)	34 (21.0)
Prevalent hyperlipidaemia^a^, *n* (%)	23 (11.6)	28 (17.3)
Medication for hyperlipidaemia^a^, *n* (%)	28 (14.1)	22 (13.6)
Prevalent cardiovascular disease^a^, *n* (%)	23 (11.6)	21 (13.0)
*Primary outcome*		
Diet quality, scored 0 (low) to 150 (high), mean (SD)	106.0 (18.3)	103.9 (19.4)
*Secondary outcomes*		
*Cardiometabolic measures, mean (SD):*		
HbA1c^b^, mmol/mol	37.2 (6.6)	37.8 (8.4)
LDL-cholesterol^c^, mmol/L	3.0 (1.0)	3.1 (0.9)
HDL-cholesterol^d^, mmol/L	1.5 (0.4)	1.6 (0.5)
Total cholesterol^e^, mmol/L	5.3 (1.5)	5.4 (1.1)
Total cholesterol/HDL-ratio^e^	3.7 (1.2)	3.6 (1.3)
Triglycerides^f^, mmol/L	1.8 (0.9)	1.8 (0.9)
Waist circumference females^g^, cm	94.6 (13.8)	93.1 (13.4)
Waist circumference males^h^, cm	104.1 (12.8)	101.9 (10.1)
Total percentage healthier food purchasing^i^	47.7 (21.7)	47.2 (22.9)
Total customer satisfaction^j^, scored 1 (low) to 7 (high), mean (SD)	5.5 (1.2)	5.8 (1.0)
*Food-decision styles for vegetables*^*k*^*, scored 1 (low) to 7 (high), mean (SD):*		
Reflective	5.3 (1.2)	5.2 (1.1)
Habitual	5.0 (0.9)	4.9 (1.0)
Impulsive	3.4 (1.2)	3.5 (1.2)
*Food-decision styles for snacks*^*l*^*, scored 1 (low) to 7 (high), mean (SD):*		
Reflective	4.1 (1.5)	3.8 (1.3)
Habitual	3.2 (1.3)	3.3 (1.4)
Impulsive	3.6 (1.6)	3.7 (1.5)
*Nudges and social cognitive factors*^*j*^*, scored 1 (low) to 7 (high), mean (SD):*		
Health goals	6.4 (0.9)	6.3 (0.8)
Healthy shopping	5.9 (1.0)	6.1 (0.9)
Perceived social norm	4.7 (0.9)	4.7 (1.0)
Attractiveness healthy foods	5.9 (1.1)	5.9 (1.2)

Low educational attainment, no education and primary education, Medium educational attainment, secondary educational attainments, High educational attainment, tertiary educational attainments.

^a^*n* = 1 missing value

^b^*n* = 40 missing values

^c^*n* = 72 missing values

^d^*n* = 57 missing values

^e^*n* = 71 missing values

^f^*n* = 63 missing values

^g^*n* = 23 missing values

^h^*n* = 6 missing values

^i^*n* = 144 missing values

^j^*n* = 2 missing values

^k^*n* = 33 missing values

^l^*n* = 153 missing values

In addition, 233 of the participants also completed the baseline measurement and at least one follow-up measurement of the mobile coaching app intervention, of whom 114 were randomised to the mobile coaching app plus step counter app (intervention) and 119 only to the step counter app (control). Participant characteristics were equally balanced between groups and comparable to the total study population (Additional file 1: Table S4).

#### Primary outcome

On average over the 12-month intervention period (i.e. combining different time points), the nudging and pricing strategies did not affect diet quality (*β* − 1.1 (95% CI − 3.8 to 1.7)) compared to the control arm (Table 3). The ICCs for clustering of individual-level data and supermarket-level data were 0.39 and 0.04, respectively. Results were comparable when analysing diet quality at T1 (*β* − 0.8 (95% CI: − 3.7 to 2.2), *n* = 345) or T2 (*β* − 0.9 (95% CI: − 3.8 to 2.1), *n* = 357).

**Table 3:** Average changes in intervention participants over six to 12 months, compared to controls (*n*_total_ = 361)

	*β* (95% CI)
*Primary outcome*	
Diet quality, scored 0 (low) to 150 (high)	− 1.1 (− 3.8 to 1.7)
*Secondary outcomes*	
*Cardiometabolic measures:*	
HbA1c^a^, mmol/mol	0.6 (− 0.1 to 1.3)
LDL-cholesterol^b^, mmol/L	− 0.0 (− 0.2 to 0.1)
HDL-cholesterol^c^, mmol/L	− 0.0 (− 0.1 to 0.0)
Total cholesterol^d^, mmol/L	0.0 (− 0.2 to 0.2)
Total cholesterol/HDL-ratio^d^	0.1 (− 0.1 to 0.2)
Triglycerides^e^, mmol/L	0.1 (− 0.1 to 0.3)
Waist circumference females^f^, cm	0.7 (− 0.8 to 2.2)
Waist circumference males^g^, cm	0.5 (− 2.4 to 3.5)
Total percentage healthier food purchasing^h^	0.7 (− 2.7 to 4.0)
Total customer satisfaction^i^, scored 1 (low) to 7 (high)	**0.3 (0.1** to** 0.5)**
*Food-decision styles for vegetables*^j^*, scored 1 (low) to 7 (high):*	
Reflective	− 0.1 (− 0.2 to 0.1)
Habitual	− 0.1 (− 0.2 to 0.1)
Impulsive	0.0 (− 0.2 to 0.2)
*Food-decision styles for snacks*^*k*^*, scored 1 (low) to 7 (high)*	
Reflective	− 0.1 (− 0.4 to 0.2)
Habitual	0.2 (− 0.0 to 0.4)
Impulsive	0.1 (− 0.2 to 0.4)
*Nudges and social cognitive factors*^*i*^*, scored 1 (low) to 7 (high):*	
Health goals	− 0.1 (− 0.3 to 0.1)
Healthy shopping	0.1 (− 0.1 to 0.3)
Perceived social norm	− 0.1 (− 0.2 to 0.1)
Attractiveness healthy foods	− 0.2 (− 0.3 to 0.0)

Analyses were based on linear mixed models including random intercepts on the participant and on the supermarket-level. All analyses are adjusted for the baseline value of the outcome of interest and time as categorical variable. Bold values represent statistically significant findings (*p* < 0.05).

^a^*n* = 40 missing values

^b^*n* = 72 missing values

^c^*n* = 57 missing values

^d^*n* = 71 missing values

^e^*n* = 63 missing values

^f^*n* = 23 missing values

^g^*n* = 6 missing values

^h^*n* = 144 missing values

^i^*n* = 2 missing values

^j^*n* = 33 missing values

^k^*n* = 153 missing values

#### Secondary outcomes

There was no significant effect of nudging and pricing strategies on the total percentage of healthy purchasing over time (*β* 0.7 (95% CI: − 2.7 to 4.0)) nor on HbA1c (*β* 0.6 (95% CI: − 0.1 to 1.3)), lipid profile (LDL *β* − 0.0 (95% CI: − 0.2 to 0.1), HDL *β* − 0.0 (95% CI: − 0.1 to 0.0)), waist circumference (females *β* 0.7 (95% CI: − 0.8 to 2.2), males *β* 0.5 (95% CI: − 2.4 to 3.5)), the food-decision styles, and all social cognitive factors related to nudges (Table 3). The mobile coaching app intervention did not significantly change daily step count (*β* − 124.0 (95% CI: − 723.1 to 475.1), *n* = 233) compared to the control arm.

Participants in the intervention arm scored 0.3 points (95% CI: 0.1 to 0.5) higher on overall customer satisfaction, compared to the control arm (Table 3). After the trial, 39% of participants from intervention supermarkets indicated having noticed changes in the supermarket lay-out, compared to 24% of control participants (Additional file 1: Table S5).

#### Sensitivity analyses

Descriptive statistics of medication use per time point indicated no notable differences between arms over time (Additional file 1: Table S6). The descriptive statistics of the diet quality scores by food group and of the total percentages of healthier food purchasing from various food groups by time point suggested no underlying trends towards potential healthier consumption of different food groups or purchases (Additional file 1: Tables S7 and S8).

Age modified the association between the intervention and diet quality (*β*_intervention group x age_ 0.16 (90% CI 0.01 to 0.31)). Nudging and pricing strategies resulted in higher diet quality with increasing age levels, as the oldest participants (80 years) in the intervention arm improved their diet quality with approximately 2 points more on average over time compared to those in the control arm (Additional file 2: Figure S2). Those youngest participants (30 years) reduced their diet quality with approximately 6 points on average over time compared to the control arm. Interaction terms with educational attainment and sex were not significant. For secondary outcomes, age modified the intervention effect on waist circumference among females (*β*_intervention group x age_ − 0.17 (90% CI − 0.29 to − 0.05)), as nudging and pricing strategies resulted in a lower waist circumference at increasing age levels (Additional file 2: Figure S3). An interaction with age was found in similar direction for the total to HDL cholesterol ratio (*β*_intervention group x age_ − 0.01 (90% CI − 0.03 to − 0.00)) (Additional file 2: Figure S4).

### Supermarket-level results

Average pre-intervention sales of the total percentage of healthy products were 27.4% (SD 2.5) in control supermarkets and 27.5% (SD 2.1) in intervention supermarkets (Additional file 1: Table S9). Compared to control supermarkets and to pre-intervention sales trends, nudging and pricing strategies did not change the total percentage of healthy products sales (*β* − 0.0 (95% CI − 0.0 to 0.0)) nor the percentage of healthy products sales within various food groups (Table 4). Nudging and pricing strategies also did not significantly change retailer revenue (*β* − 76.9 Euros (95% CI − 274.6 to 120.4)). Results were comparable when excluding the intervention supermarket with the lowest implementation fidelity score over time (*β*_total percentage of healthy sales_ 0.0 (95% CI − 0.0 to 0.1)).

**Table 4:** Average weekly changes in intervention supermarkets over six to 12 months, compared to controls (*n* = 12)

	*β* (95% CI)
Total percentage of healthy product sales	− 0.0 (− 0.0 to 0.0)
*Percentage change within various food groups:*	
Healthy fruits, vegetables, legumes and nuts sales	− 0.0 (− 0.1 to 0.1)
Healthy grain product sales	− 0.0 (− 0.1 to 0.1)
Healthy milk and yogurt products sales	− 0.0 (− 0.1 to 0.1)
Healthy cheese sales	0.0 (− 0.1 to 0.1)
Healthy meat products, meat substitutes and egg sales	− 0.0 (− 0.1 to 0.1)
Healthy fish sales	0.0 (− 0.1 to 0.1)
Healthy oils, fats and herbs and spices sales	0.0 (− 0.1 to 0.1)
Healthy non-alcoholic beverage sales	0.1 (− 0.1 to 0.2)
Healthy product sales from remaining food products	− 0.0 (− 0.1 to 0.0)
Unhealthy sweet and savoury snack sales of total sales	− 0.0 (− 0.0 to 0.0)
Total sales revenue (Euros)	− 76.9 (− 274.6 to 120.4)

Analyses were based on controlled interrupted time series analyses using linear mixed models, treating the 6 months pre-intervention sales trends by supermarket as a fixed effect and including a random intercept for supermarkets

The median implementation fidelity score for all supermarkets over the intervention period was 3.6 (range 2.9–4.1) which translates to an implementation fidelity of 72%. Mean scores for individual supermarkets by time point are presented in Additional file 2: Figure S5.

## Discussion

This cluster-RCT did not show effectiveness of a combination of real-world nudging and pricing strategies promoting healthier purchasing across a wide range of product groups on diet quality and food purchases nor on cardiometabolic risk markers. We found a small positive effect on customer satisfaction. Supermarket sales data trends were unaffected following implementation of nudging and pricing strategies, confirming the individual-level results. The mobile coaching app did not change daily step counts.

Strengths of this study include testing a combination of nudging and pricing strategies implemented across multiple food groups, the cluster-randomised and controlled design with long follow-up, the real-world nature of the data, outcome measurement of both diet quality and purchase data, and verification of individual-level results with supermarket-level results. Some limitations should be acknowledged. Quantitative questionnaires are prone to over- and underreporting and socially desirable answers. While the short 40-item FFQ minimised participant burden, it is less sensitive for detecting changes over time compared to a full-length FFQ. Although the co-creative intervention development and the supermarket-led implementation enabled a sustainable and scalable intervention, it probably also resulted in the interventions having less impact, with a stronger focus on nudging than on pricing strategies, and on promoting healthy versus discouraging unhealthy products. Despite recruiting in socially disadvantaged neighbourhoods, a relatively high proportion of participants had higher educational attainment and a relatively high diet quality score at baseline. However, potential attenuation of intervention effects due to selection bias is unlikely since the supermarket-level results, which reflect the intervention effects for all types of customers, confirmed the individual-level results. Last, since this trial was conducted during the COVID-19 pandemic, attenuation of potential intervention effects due to changed grocery shopping behaviours following the pandemic cannot be ruled out.

Thus far, only two comparable real-world studies have investigated a combination of nudging and pricing strategies, by targeting a specific food group. In contrast to our findings, these studies showed positive effects of combined placement, availability, and pricing interventions on peanuts sales at check-outs and on fruit and vegetable sales [33, 34]. Our null results might be explained by the relatively low intervention dosage of pricing strategies (targeting a mere 3% of the supermarket assortment according to a dynamic implementation pattern) and overall moderate implementation fidelity. The co-creation process showed that the supermarket chain was only willing to implement low risk interventions as more drastic changes (e.g. placing white bread behind a counter, or more extensive pricing strategies) were perceived to threaten profit margins and market position [43]. For pricing strategies to be effective, they may need to be structurally implemented within a food group (or multiple food groups) as a whole.

Other RCTs and non-randomised controlled trials, investigating nudges as single intervention components while using real-world purchasing, sales, or intake data, showed mixed effects. When significant, effect sizes were generally modest around 1–2% [64–75]. For pricing interventions, effects were more convincing [76–81]. More drastic nudge approaches would likely yield large(r) effect sizes (e.g. 2% increase in fruit and vegetable sales after relocating the fruits and vegetable section to a prominent location [75]) than, for example, an information nudge (e.g. 0.6% increase in healthy sales following shelf labels) [69]. Lessons from our and other real-world supermarket trials suggest that price discounts are effective and thus have the potential to improve diet quality, but only if applied structurally across enough food groups and when combined with price increases to maximise impact [76–81]. Nudging strategies are promising, but in real-world settings, they need to be drastic or multiple in order to sort a substantial effect [65, 66, 69–75]. In addition, nudging and pricing strategies may need to be combined to optimise their effectiveness [22, 33, 34]. Finally, our and other studies predominantly focused on stimulating healthier purchases, while mostly leaving unhealthy products untargeted. Considering that the majority of the supermarket assortment can be considered unhealthy [13], this leaves a large proportion of the supermarket assortment untargeted by interventions. Our findings in combination with the mixed evidence base on real-world studies suggests that unhealthy products also need to be targeted by interventions to exert an impact on diet quality.

The mobile coaching app did not change daily step counts. In a separate paper, we will present further exploratory findings suggesting effect modification by users’ perceived usefulness of the app. The lack of the main effect contrasts a meta-analysis of RCTs reporting a ~ 1500 step increase in daily step count with mobile interventions [32]. In our study, the app design did not allow for monitoring whether participants read the messages, but it might be that insufficient exposure to coaching messages led to the lack of effect on step counts [82]. Future studies should thus consider measuring user engagement.

### Implications

Our results demonstrate that co-creation of interventional strategies with a retailer led to a predominant focus on the promotion of healthy products in supermarkets, which appeared not to be sufficient to shift purchasing patterns and dietary intake towards healthier patterns. Nevertheless, nudging and pricing strategies can be part of a comprehensive set of ambitious policy measures, while simultaneously focusing on discouraging unhealthier purchasing (e.g. restricting marketing and promotions of unhealthy products, banning of unhealthy products at checkouts, and restricting the availability of unhealthy products) [83, 84]. Such a combination of policy measures would create a level playing field among food retailers which is crucial in overcoming commercial barriers that impede impactful changes in supermarket environments. Furthermore, combined policy measures can lead to a small shift in population diet quality which can have a positive impact on population health outcomes [11]. Implementation fidelity and its effects on purchasing patterns needs to be monitored, and measures should be reformulated based on emerging insights.

There is a need for further studies focusing on the healthiness of purchase patterns or dietary intake patterns as a whole, rather than on single products or product groups, to better estimate the intervention implications for public health. Researchers may consider using more flexible study designs for real-world contexts that aim to generate public health benefits; various types of intervention components can be implemented simultaneously and innovated over a trial period based on real-time evaluations of their effectiveness [85]. Such an adaptive design could increase the chances of developing the most effective intervention(s), with the highest likelihood of achieving sustainable effects.

## Conclusions

This study showed that co-created nudging and pricing strategies, as well as mobile physical activity coaching strategies, were not effective in a real-world context in promoting healthier diets and purchasing patterns, increasing step count or in improving cardiometabolic health. Focussing on the promotion of healthy products predominantly via nudging strategies targeting healthy products and some pricing strategies appears to be insufficient to counterbalance the high availability and promotion of unhealthy products in supermarkets. Comprehensive policy measures are essential to establish a level playing field among food retailers, focusing both on discouraging the purchase of unhealthy products and promoting healthier options. These policies should ensure fair competition among retailers while simultaneously working towards improving population diets and preventing cardiometabolic diseases.

### Supplementary Information

**Additional file 1: Tables S1-S9.** Table S1—Available number and type of healthy and unhealthy products per food group on average across participating supermarkets (*n* = 12); Table S2—Intervention dosage of pricing strategies across food groups over the trial period; Table S3—Individual-level outcomes and their measurement methods; Table S4—Population characteristics and baseline measurements of participants of the mobile coaching app intervention in the Supreme Nudge trial (*n* = 233), stratified by mobile coaching app arm; Table S5—Descriptive statistics of nudge appreciation and nudge awareness during the last follow-up measurement at month 6 or month 12 (*n* = 332); Table S6—Descriptive statistics of medication use per time point (*n* = 361), stratified by trial arm; Table S7—Descriptive statistics per time point of the diet quality scores per food group (*n* = 361), stratified by trial arm; Table S8—Descriptive statistics per time point of the total percentage of healthy purchasing from different food groups (*n* = 217), stratified by trial arm; Table S9—Average pre-intervention and post-intervention sales per week for all intervention and all control supermarkets for each supermarket-level outcome (*n* = 12).

**Additional file 2: Figures S1-S5.** Figures S1a-k—Photo examples of nudge intervention: Figure S2—Average diet quality scores over time for different age levels in the intervention group and the control group of the Supreme Nudge trial (*n* = 361); Figure S3—Average waist circumference among females over time for different age levels in the intervention group and the control group of the Supreme Nudge trial (*n* = 239); Figure S4—Average total cholesterol to HDL ratio over time for different age levels in the intervention group and the control group of the Supreme Nudge trial (*n* = 290); Figure S5—Mean implementation fidelity scores (range 1–5) per month over the intervention period of one year, by supermarket.

**Additional file 3.** CONSORT 2010 checklist for cluster randomised trials.

## Data Availability

The data analysed during the current study are not publicly available as this would violate participant consent (individual-level data) or confidentiality agreements (supermarket-level data). The analyses plans and analytical codes are available from the corresponding author on reasonable request.

## Abbreviations

ICC: Intra-cluster correlation coefficient
HbA1c: Glycated haemoglobin
HDL: High-density lipoprotein cholesterol
LDL: Low-density lipoprotein cholesterol
RCT: Randomised controlled trial
SEP: Socioeconomic position
