# Supplementary material for: Real-world nudging, pricing, and mobile physical activity coaching was insufficient to improve lifestyle behaviours and cardiometabolic health: the Supreme Nudge parallel cluster-randomised controlled supermarket trial
Source: BMC Med. 2024 Feb 2;22:52. doi: 10.1186/s12916-024-03268-4 (PMC10835818; doi:10.1186/s12916-024-03268-4)
Supplement: Supplementary file 1 — Additional file 1: Tables S1-S9. Table S1—Available number and type of healthy and unhealthy products per food group on average across participating supermarkets (n = 12); Table S2—Intervention dosage of pricing strategies across food groups over the trial period; Table S3—Individual-level outcomes and their measurement methods; Table S4—Population characteristics and baseline measurements of participants of the mobile coaching app intervention in the Supreme Nudge trial (n = 233), stratified by mobile coaching app arm; Table S5—Descriptive statistics of nudge appreciation and nudge awareness during the last follow-up measurement at month 6 or month 12 (n = 332); Table S6—Descriptive statistics of medication use per time point (n = 361), stratified by trial arm; Table S7—Descriptive statistics per time point of the diet quality scores per food group (n = 361), stratified by trial arm; Table S8—Descriptive statistics per time point of the total percentage of healthy purchasing from different food groups (n = 217), stratified by trial arm; Table S9—Average pre-intervention and post-intervention sales per week for all intervention and all control supermarkets for each supermarket-level outcome (n = 12). [file 12916_2024_3268_MOESM1_ESM.pdf]

Real-world nudging, pricing and mobile physical activity coaching was insufficient to improve lifestyle behaviours and cardiometabolic health: the Supreme Nudge parallel cluster-randomised controlled supermarket trial

Supplementary tables

Josine M Stuber, et al

Table S1. Available number and type of healthy and unhealthy products[a] per food group on average across participating supermarkets (n=12). .. 2

Table S2. Intervention dosage of pricing strategies across food groups over the trial period. 4

Table S3. Individual-level outcomes and their measurement methods. 5

Table S4. Population characteristics and baseline measurements of participants of the mobile coaching app intervention in the Supreme Nudge trial (n=233), stratified by mobile coaching app arm. 6

Table S5. Descriptive statistics of nudge appreciation and nudge awareness during the last follow-up measurement at month 6 or month 12 (n=332). 7

Table S6. Descriptive statistics of medication use per time point (n=361), stratified by trial arm. 8

Table S7. Descriptive statistics per time point of the diet quality (Dutch Healthy Diet 2015 index) scores per food group (n=361), stratified by trial arm. 9

Table S8. Descriptive statistics per time point (blocks of four weeks) of the total percentage of healthier food purchasing from different food groups (n=217), stratified by trial arm. 11

Table S9. Average (mean (SD)) pre-intervention and post-intervention sales per week for all intervention and all control supermarkets for each supermarket-level outcome (n=12). 13

Table S1. Available number and type of healthy and unhealthy products[a] per food group on average across participating supermarkets (n=12).

	Healthy products[b]	Unhealthy or neutral products
All foods and beverages (*n*≈12,641)	*n*≈2,368 (18.7% of all products)	*n*≈10,273
Fruits (*n*≈241)	Fresh, pre-cut and frozen fruits, canned fruits, apple sauce (*n*≈180; 75% of all healthy fruits)	Canned fruits with added sugar, apple sauce with added sugar, fruit puree with added sugar (*n*≈61)
Vegetables (*n*≈643)	Fresh, pre-cut and frozen vegetables, fresh herbs , canned vegetables (*n*≈395; 61% of all healthy vegetables),	Vegetables with added cream, canned vegetables (salted) (*n*≈248)
Breads (*n*≈243)	Wholegrain bread (*n*≈88; 36% of all healthy bread)	White and non-wholegrain brown bread (*n*≈155)
Bread substitutes (*n*≈302)	Wholegrain crackers, oats, muesli, and breakfast cereals with low added sugar, salt and fat (*n*≈67; 22% of all healthy bread substitutes)	Non-wholegrain crackers, breakfast cereals with added sugar, salt and/or fat, rice crackers, rusk (*n*≈235)
Potatoes (*n*≈137)	Fresh and/or pre-cut unprocessed potatoes (n≈66; 48% of all healthy potatoes)	Processed potatoes, mashed potato mix (*n*≈71)
Pasta and rice (*n*≈268)	Wholegrain pasta, rice and wraps (*n*≈44; 16% of all healthy pasta and rice)	Non-wholegrain (refrigerated) pasta, rice, wraps, couscous (*n*≈224)
Tea and coffee (*n*≈496)	Tea bags, filtered coffee products, coffee products with semi-skimmed and skimmed dairy (*n*≈420; 85% of all healthy tea and coffee)	Unfiltered coffee products, tea bags (added sugar) (*n*≈76), coffee with added sugar or full-fat dairy
Sodas, waters and juices (*n*≈915)	Water, flavoured water (unsweetened) (*n*≈72; 8% of all healthy beverages)	Sodas, energy drinks, fruit juices, lemonade (*n*≈843)
Cheese (*n*≈444)	Low-fat and low-salt cheeses (*n*≈66; 15% of all healthy cheese)	High-fat and/or high-salt cheeses (*n*≈378)
Milk and yogurt products (*n*≈680)	Semi-skimmed and skimmed milk and yogurt products, plant-based dairy products (unsweetened), coffee milk (*n*≈190; 28% of all healthy milk and yogurt products)	Semi-skimmed and skimmed dairy products (sweetened), full-fat dairy, custard, desserts, pudding, whipped cream, pudding, cooking cream, dairy drinks, chocolate milk, soy-dairy products (sweetened), (*n*≈490)
Meat products (*n*≈1,024)	Unprocessed and low-fat meats, plant-based meat substitutes (unsalted), and eggs (*n*≈102; 10% of all healthy meat products)	Processed and high-fat meats, and plant-based meat substitutes (salted) (*n*≈922)
Fish (*n*≈249)	Fresh, frozen, canned and breaded fish (>70% fish) (*n*≈176; 71% of all healthy fish)	Breaded fish (<70% fish), processed fish dishes (*n*≈73)
Legumes (*n*≈158)	Canned legumes (low salt) (*n*≈95; 60% of all healthy legumes)	Canned legumes (high salt) (*n*≈63)
Nuts (*n*≈209)	Seeds, nuts, peanuts, natural peanut butter (*n*≈77; 37% of all healthy nuts)	Nuts, peanuts (salted or sugared), peanut butter (added salt and palm oil) (*n*≈132)
Fats (*n*≈190)	Olive oils, sunflower oils, margarines, vegetable oils, frying oils (*n*≈125; 66% of all healthy fats)	Butters, baking butters, frying oils, coconut oils, butters, baking butters (*n*≈65)
Other foods (*n*≈2,836)	Whole-grain flower, yeast, dried herbs/spices, vinegar (n≈204; 7% of all healthy other foods)	Ready-to-eat meals, meal salads, pancakes, pizza, canned soup, drinking broth, seasoning products, sauces (refrigerated), fresh dough, bread toppings, spreads, baking mixes, sugar (*n*≈2,632)
Savoury snacks (*n*≈552)	N/A	Salty snacks, fried snacks, chips, popcorn, (*n*≈552)

Sweet snacks (*n*≈1,822)	N/A	Cookies, candy, confectionary, chocolate, liquorice, bubble-gum, ice cream (water- and milk based) (*n*≈1,822)
Alcoholic drinks (*n*≈1,231)	N/A	All alcoholic drinks (*n*≈1,231)

[a]Absolute number of products per food group is indicative as the product assortment varies across supermarket locations and is subjected to weekly changes.

[b]Healthy products were classified as those which are recommended within in the Dutch dietary guidelines (Kromhout et al (2016) EJCN).

N = number; N/A = Not applicable.

Table S2. Intervention dosage of pricing strategies across food groups over the trial period.

Food group	Price decrease			Price increase		
	Weeks targeted (%)	N average products targeted per week -15%	N average products targeted per week -25%	Weeks targeted (%)	N average products targeted per week +10%	N average products targeted per week +15%
Fruits	48 (85.7%)	N/A	12,3	N/A	N/A	N/A
Vegetables	49 (87.5%)	12,3 (7 weeks)	21,1 (49 weeks)	3 (5,4%)	19 (3 weeks)	N/A
Breads	31 (55.4%)	12.6 (25 weeks)	12.7 (6 weeks)	25 (44,6%)	17.2 (25 weeks)	N/A
Bread substitutes	31 (55.4%)	16.0 (31 weeks)	N/A	33 (58.9%)	67.6 (33 weeks)	N/A
Potatoes	N/A	N/A	N/A	N/A	N/A	N/A
Pasta and rice	17 (30.4%)	12.5 (17 weeks)	N/A	17 (30.4%)	60.0 (17 weeks)	N/A
Teas and coffees	8 (14.3%)	99.8 (4 weeks)	48.0 (4 weeks)	4 (7.1%)	5 (4 weeks)	N/A
Sodas, waters and juices	22 (39.3%)	45.7 (13 weeks)	24.0 (11 weeks)	21 (37.5%)	83.5 (10 weeks)	93.3 (11 weeks)
Cheeses	12 (21.4%)	29.0 (12 weeks)	N/A	12 (21.4%)	82.7 (12 weeks)	N/A
Milk and yogurt products	9 (16.1%)	28.1 (9 weeks)	N/A	16 (28.6%)	67.4 (9 weeks)	58.1 (7 weeks)
Meats	N/A	N/A	N/A	N/A	N/A	N/A
Fish	20 (35.7%)	14.5 (8 weeks)	30.1 (16 weeks)	8 (14,3%)	28.0 (8 weeks)	N/A
Legumes	16 (28.6%)	N/A	28.5 (16 weeks)	N/A	N/A	N/A
Nuts	19 (33.9%)	25.9 (16 weeks)	N/A	19 (33.9%)	38.2 (19 weeks)	N/A
Fats	26 (46.4%)	43.8 (22 weeks)	29.0 (4 weeks)	19 (33.9%)	50.6 (19 weeks)	N/A
Other foods	4 (7.1%)	N/A	9 (4 weeks)	7 (12.5%)	9.4 (7 weeks)	N/A
Savoury snacks	N/A	N/A	N/A	11 (19.6%)	21.5 (11 weeks)	N/A
Sweet snacks	N/A	N/A	N/A	16 (28.6%)	3.5 (16 weeks)	N/A
Alcoholic drinks	N/A	N/A	N/A	N/A	N/A	N/A

N = number; N/A = Not applicable.

Table S3. Individual-level outcomes and their measurement methods.

Outcomes	Operationalisation	Measurement method	Time points
Population characteristics			
Age	Age in years at time of study registration.	Baseline questionnaire.	T0.
Sex	Male; female.	Baseline questionnaire.	T0.
Highest completed educational level	Low educational attainment (no education, primary school); medium educational attainment (secondary educational attainments); high educational attainment (tertiary/higher educational attainments).	Baseline questionnaire.	T0.
Household size	Number of adults; number of children.	Baseline questionnaire.	T0.
Smoking status	Current smoker; irregular smoker; former smoker; never smoked.	Baseline questionnaire.	T0.
Medical history	Prevalent type 2 diabetes; hypertension; hyperlipidaemia; cardiovascular disease (all: yes, no, do not know).	Baseline questionnaire.	T0.
Medication use	For type 2 diabetes; hypertension; hyperlipidaemia (all: yes, no, do not know).	Baseline questionnaire and follow-up questionnaires.	T0, T1, T2, T3.
Primary outcome			
Total dietary guideline adherence	Scored 0 (low adherence) to 150 (high adherence).	Short 40-item food frequency questionnaire.	T0, T1, T2, T3.
Secondary outcomes			
Cardiometabolic measures	HbA1c (mmol/mol); LDL-cholesterol (mmol/L); HDL-cholesterol (mmol/L); total cholesterol (mmol/L); total cholesterol/HDL-ratio; triglycerides (mmol/L); waist circumference (cm).	Ah-home measurements by participants, via a finger prick and a measuring tape.	T0, T2, T3.
Total percentage healthier food purchasing	Total percentage of healthy product purchases based on the total grams or millilitres purchased per block of four weeks to reflect an average purchasing pattern.	Supermarket loyalty card.	Complete study period (weekly data).
Step counts	Daily step counts extracted from the smartphones' pedometer or accelerometer.	The 'SNapp' step-counter app.	Complete study period (daily data).
Total customer satisfaction	Overall customer satisfaction, measured on a 7-point Likert scale (very unsatisfied – very satisfied).	Baseline questionnaires and all follow-up questionnaires.	T0, T1, T2, T3.
Food decision styles	Reflective; habitual; impulsive (for vegetables and for snacks i.e. sweets and cakes), measured on a 7-point Likert scale (strongly disagree – strongly agree).	Baseline questionnaires and all follow-up questionnaires.	T0, T1, T2, T3.
Nudges and social cognitive factors	Health goals; healthy shopping; perceived social norm; attractiveness healthy foods, measured on a 7-point Likert scale (strongly disagree – strongly agree)	Baseline questionnaires and all follow-up questionnaires.	T0, T1, T2, T3.
Acceptance and awareness of nudges	Nudge appreciation (i.e., "appreciation of help with making healthier choices" measured on a 7-point Likert scale (strongly disagree – strongly agree)), nudge awareness (i.e., noticed any changes in store lay-out: yes/no, if yes: describe changes (open-end)).	Follow-up questionnaire at month 6 or 12.	T0, T1, T2, T3.

N/A = not applicable. Time points = baseline (T0), after 3 months (T1), after 6 months (T2), and after 12 months (T3).

Table S4. Population characteristics and baseline measurements of participants of the mobile coaching app intervention in the Supreme Nudge trial (n=233), stratified by mobile coaching app arm.

	Intervention (n=114)		Control (n=119)	
Age (years), mean (*SD*)	55.9	(11.3)	57.0	(10.7)
Gender (female) [a], *n* (%)	84	(73.7)	87	(73.1)
Educational level, *n* (%)				
Low	1	(0.9)	2	(1.7)
Medium	65	(57.0)	64	(53.8)
High	48	(42.1)	53	(44.5)
Operating system, *n* (%):				
Android	75	(65.8)	84	(70.6)
iOS	39	(34.2)	35	(29.4)
Sensor type [b], *n* (%):				
Step detector	8	(7.0)	22	(18.5)
Step counter	105	(92.1)	97	(81.5)
Baseline daily step count, median [IQR][a]	3356	[3871]	3008	[3588]

Low educational attainment: no education and primary education; Medium educational attainment: secondary educational attainments; High educational attainment: tertiary educational attainments. [a]n=2 missing values; [b]n=1 missing value.

Table S5. Descriptive statistics of nudge appreciation and nudge awareness during the last follow-up measurement at month 6 or month 12 (n=332).

	Control supermarkets (n=176)	Intervention supermarkets (n=156)
Nudge appreciation, scored 1 (low) to 7 (high), mean (SD)		
Appreciation of help with making healthier choices	5.1 (1.7)	4.8 (1.8)
Noticed changes in store lay-out, n (%)		
Yes	43 (24.4)	61 (39.1)
No	133 (75.6)	95 (60.9)
If 'yes' on noticed changes, noticed changes were, n (%)		
Nudges were perceived to be noticed	5 (11.6)	16 (26.2)
Other changes were noticed (e.g., store renovation)	32 (74.4)	39 (63.9)
Unclear if nudges were noticed	6 (14.0)	6 (9.8)
Perceived noticed nudges, n (%)		
Healthy checkout	1 (20.0)	9 (56.3)
Healthy end of aisle promotions	0 (0.0)	2 (12.5)
Increased noticeability/findability of healthy products	2 (40.0)	2 (12.5)
Increased fruit and vegetable promotions	1 (20.0)	2 (12.5)
Healthier shelf arrangement	1 (20.0)	0 (0.0)
Aisle baskets with healthy products	0 (0.0)	1 (6.3)

Table S6. Descriptive statistics of medication use per time point (n=361), stratified by trial arm.

	Time point	Control supermarkets (n=199)		Intervention supermarkets (n=162)	
Medication for type 2 diabetes, n (%)	T0	14	(7.0%)	8	(4.9%)
	T1	13	(6.5%)	9	(5.6%)
	T2[a]	11	(5.7%)	7	(4.3%)
	T3[b]	10	(7.8%)	7	(7.4%)
Medication for hypertension, n (%)	T0	37	(18.6%)	34	(21.0%)
	T1	40	(20.1%)	34	(21.0%)
	T2[a]	37	(19.3%)	36	(22.2%)
	T3[b]	33	(25.8%)	22	(23.4%)
Medication for hyperlipidaemia, n (%)	T0	28	(14.1%)	22	(13.6%)
	T1	25	(12.6%)	27	(16.7%)
	T2[a]	27	(14.1%)	28	(17.3%)
	T3[b]	24	(18.8%)	15	(16.0%)

T0 = baseline; T1 = after 3 months; T2 = after 6 months; T3 = after 12 months.
[a]n=7 missing values; [b]n=139 missing values.

Table S7. Descriptive statistics per time point of the diet quality (Dutch Healthy Diet 2015 index) scores per food group (n=361), stratified by trial arm.

	Time point	Control supermarkets (n=199)		Intervention supermarkets (n=162)	
Healthy intake of vegetables (score 0-10), mean (SD)	T0	6.4	(3.2)	5.9	(3.2)
	T1[a]	6.5	(3.2)	6.0	(3.1)
	T2[b]	6.2	(3.0)	5.5	(3.0)
	T3[c]	5.8	(3.0)	5.3	(3.1)
Healthy intake of fruits (score 0-10), mean (SD)	T0	7.0	(3.2)	6.4	(3.4)
	T1[a]	7.1	(3.2)	6.8	(3.3)
	T2[b]	7.0	(3.2)	6.4	(3.4)
	T3[c]	6.9	(3.3)	6.6	(3.3)
Healthy intake of whole grains (score 0-10), mean (SD)	T0	7.5	(2.5)	7.4	(2.8)
	T1[a]	7.4	(2.9)	7.4	(2.9)
	T2[b]	7.7	(2.7)	7.5	(2.9)
	T3[c]	8.0	(2.6)	7.1	(3.2)
Healthy intake of legumes (score 0-10), median [IQR]	T0	10.0	[7.8]	8.7	[10.0]
	T1[a]	10.0	[5.6]	10	[10.0]
	T2[b]	8.7	[5.6]	8.7	[10.0]
	T3[c]	10.0	[5.6]	10.0	[4.6]
Healthy intake of nuts (score 0-10), median [IQR]	T0	5.8	[8.1]	3.9	[8.1]
	T1[a]	5.8	[8.1]	2.9	[8.1]
	T2[b]	5.8	[8.1]	2.9	[7.7]
	T3[c]	6.8	[8.1]	2.9	[9.0]
Healthy intake of dairy (score 0-10), median [IQR]	T0	6.0	[6.1]	5.7	[6.8]
	T1[a]	5.6	[6.5]	5.4	[5.8]
	T2[b]	6.0	[6.4]	5.3	[6.5]
	T3[c]	6.4	[6.0]	6.3	[5.3]
Healthy intake of fish (score 0-10), median [IQR]	T0	7.5	[7.5]	7.5	[7.5]
	T1[a]	7.5	[6.2]	7.5	[7.5]
	T2[b]	7.5	[6.2]	7.5	[7.5]
	T3[c]	7.5	[6.2]	7.5	[7.5]
Healthy intake of tea (score 0-10), median [IQR]	T0	3.3	[9.8]	5.0	[9.8]
	T1[a]	5.2	[9.8]	5.2	[9.8]
	T2[b]	3.3	[9.8]	5.2	[9.8]
	T3[c]	5.2	[9.5]	3.3	[10.0]
Healthy intake of oils and butter (score 0-10), median [IQR]	T0	10.0	[9.7]	10.0	[9.6]
	T1[a]	10.0	[9.7]	10.0	[9.1]
	T2[b]	10.0	[9.7]	10.0	[9.7]
	T3[c]	10.0	[9.7]	10.0	[8.5]
Healthy intake of coffee (score 0-10), mean (SD)	T0	7.4	(2.8)	8.1	(2.6)
	T1[a]	7.3	(2.8)	7.9	(2.6)
	T2[b]	7.5	(2.7)	7.8	(2.6)
	T3[c]	7.3	(2.8)	7.6	(2.7)
Healthy/limited intake of red meat (score 0-10), mean (SD)	T0	8.7	(2.8)	9.1	(2.4)
	T1[a]	9.3	(2.1)	9.3	(2.2)
	T2[b]	9.3	(2.2)	9.2	(2.4)
	T3[c]	9.0	(2.5)	9.4	(1.9)
Healthy/limited intake of processed meat (score 0-10), median [IQR]	T0	5.1	[6.9]	5.8	[5.5]
	T1[a]	5.5	[6.0]	6.4	[5.0]
	T2[b]	5.1	[7.3]	6.5	[5.0]
	T3[c]	5.8	[6.1]	5.9	[5.0]
Healthy/limited intake of sugar sweetened beverages (score 0-10), mean (SD)	T0	8.2	(3.0)	7.9	(3.2)
	T1[a]	8.4	(2.8)	8.0	(3.3)
	T2[b]	8.4	(2.7)	8.2	(2.9)
	T3[c]	8.4	(2.7)	7.9	(3.0)
Healthy/limited intake of alcoholic beverages (score 0-10), mean (SD)	T0	8.3	(2.9)	7.6	(3.6)
	T1[a]	8.2	(3.1)	7.5	(3.7)
	T2[b]	8.3	(2.8)	7.9	(3.4)
	T3[c]	8.5	(2.6)	8.1	(3.3)

Healthy/limited intake of salt (score 0-10), mean (SD)	T0	8.1	(2.1)	8.4	(2.0)
	T1[a]	8.5	(2.0)	8.6	(1.8)
	T2[b]	8.3	(2.2)	8.6	(1.8)
	T3[c]	8.3	(2.2)	8.6	(1.8)
Healthy/limited intake of unhealthy snacks (score 0-10), mean (SD)	T0	4.0	(4.2)	4.1	(4.3)
	T1[a]	4.7	(4.4)	4.4	(4.3)
	T2[b]	4.7	(4.3)	4.8	(4.4)
	T3[c]	4.9	(4.4)	4.6	(4.3)

T0 = baseline; T1 = after 3 months; T2 = after 6 months; T3 = after 12 months.
[a]n=16 missing values; [b]n=33 missing values; [c]n=148 missing values

Table S8. Descriptive statistics per time point (blocks of four weeks) of the total percentage of healthier food purchasing from different food groups (n=217), stratified by trial arm.

	Time point	Control supermarkets (n=123)		Intervention supermarkets (n=94)	
Total percentage of healthy fruits, vegetables, legumes and potato, mean (SD)	T0	85.4	(22.0)	82.0	(26.8)
	T1[b]	89.4	(14.3)	88.2	(19.7)
	T2[c]	82.9	(23.9)	84.3	(22.8)
	T3[d]	85.6	(23.0)	86.1	(22.4)
	T4[e]	85.6	(24.0)	86.4	(21.2)
	T5[f]	82.7	(28.3)	88.3	(19.4)
	T6[g]	86.1	(21.9)	81.7	(29.1)
	T7[h]	85.0	(22.9)	84.9	(25.6)
	T8[i]	85.3	(23.1)	83.7	(24.8)
	T9[j]	88.4	(16.6)	75.9	(29.5)
	T10[k]	82.7	(24.9)	86.1	(18.4)
	T11[l]	84.6	(23.2)	82.3	(19.9)
	T12[m]	86.6	(21.9)	77.3	(27.1)
	T13[n]	82.3	(29.2)	78.3	(29.6)
Total percentage of healthy grain products (bread, pasta, rice, bread substitutes, breakfast grains), median [IQR]	T0	20.9	[59.7]	25.5	[66.6]
	T1[b]	21.5	[50.0]	31.0	[59.4]
	T2[c]	22.2	[46.1]	27.2	[72.4]
	T3[d]	18.5	[50.0]	29.8	[58.3]
	T4[e]	29.3	[52.2]	20.8	[47.8]
	T5[f]	19.3	[48.7]	21.4	[54.5]
	T6[g]	23.4	[51.8]	0.0	[46.7]
	T7[h]	28.2	[51.7]	13.6	[42.7]
	T8[i]	19.4	[54.1]	6.9	[38.6]
	T9[j]	15.7	[60.0]	0.0	[46.1]
	T10[k]	22.6	[57.2]	16.0	[50.9]
	T11[l]	20.5	[55.2]	10.2	[50.5]
	T12[m]	20.7	[57.8]	25.7	[67.8]
	T13[n]	4.2	[47.0]	0.0	[33.4]
Total percentage of healthy diary (cheese, yoghurt, milk, desserts), median [IQR]	T0	44.1	[40.3]	52.1	[50.3]
	T1[b]	46.3	[38.0]	52.5	[41.9]
	T2[c]	49.7	[36.9]	45.6	[45.2]
	T3[d]	41.9	[44.4]	52.8	[47.6]
	T4[e]	47.8	[36.1]	53.7	[42.9]
	T5[f]	47.4	[50.0]	49.0	[49.7]
	T6[g]	52.3	[39.1]	51.7	[51.6]
	T7[h]	45.1	[41.8]	45.9	[64.4]
	T8[i]	43.1	[42.4]	41.9	[44.9]
	T9[j]	35.7	[49.1]	33.8	[56.1]
	T10[k]	36.3	[39.3]	40.5	[51.1]
	T11[l]	39.6	[51.3]	41.4	[47.3]
	T12[m]	46.3	[39.7]	32.4	[47.7]
	T13[n]	44.4	[44.4]	44.2	[52.2]
Total percentage of healthy fats (fish, nuts, butter and oils), median [IQR]	T0	43.1	[100.0]	43.5	[76.1]
	T1[b]	50.2	[82.6]	52.2	[78.6]
	T2[c]	52.0	[85.0]	54.2	[76.6]
	T3[d]	46.5	[81.2]	55.1	[100.0]
	T4[e]	40.0	[91.2]	68.6	[100.0]
	T5[f]	52.2	[86.4]	46.2	[93.1]
	T6[g]	38.1	[73.9]	43.3	[87.1]
	T7[h]	25.6	[73.6]	30.8	[75.0]
	T8[i]	44.4	[73.1]	33.9	[74.3]
	T9[j]	36.0	[73.5]	47.9	[81.8]
	T10[k]	46.8	[77.1]	32.1	[75.1]
	T11[l]	38.4	[80.1]	45.4	[83.1]
	T12[m]	58.3	[100.0]	54.8	[76.5]
	T13[n]	31.5	[77.0]	31.5	[76.4]

Total percentage of healthy beverages (soda, water, juices, tea, coffee), median [IQR]	T0	0.0	[15.7]	0.0	[25.1]
	T1[b]	3.5	[28.3]	7.6	[50.0]
	T2[c]	3.4	[37.4]	3.3	[58.4]
	T3[d]	0.3	[13.4]	1.9	[34.3]
	T4[e]	2.4	[50.0]	2.0	[26.7]
	T5[f]	2.2	[24.9]	0.0	[35.5]
	T6[g]	2.9	[33.1]	0.0	[28.6]
	T7[h]	0.0	[15.6]	0.0	[36.1]
	T8[i]	2.9	[20.8]	3.4	[73.4]
	T9[j]	2.3	[47.1]	0.0	[53.2]
	T10[k]	1.3	[19.7]	0.0	[48.2]
	T11[l]	1.7	[56.9]	16.1	[100.0]
	T12[m]	0.3	[58.0]	1.7	[52.2]
	T13[n]	0.0	[35.4]	0.0	[33.3]
Total percentage of snacks of all purchases (sweet and savoury snacks), median [IQR]	T0	4.0	[5.6]	4.8	[7.0]
	T1[b]	5.2	[5.2]	5.0	[7.6]
	T2[c]	5.2	[6.0]	5.1	[5.7]
	T3[d]	5.2	[5.3]	4.6	[5.9]
	T4[e]	5.1	[6.0]	5.0	[7.4]
	T5[f]	4.0	[6.2]	4.6	[5.1]
	T6[g]	4.9	[5.6]	4.3	[7.3]
	T7[h]	5.7	[6.6]	5.6	[7.0]
	T8[i]	5.6	[5.0]	5.7	[6.5]
	T9[j]	4.1	[7.1]	5.5	[4.4]
	T10[k]	4.9	[6.2]	3.7	[4.3]
	T11[l]	4.6	[7.0]	4.0	[6.0]
	T12[m]	4.9	[6.6]	4.2	[5.1]
	T13[n]	5.4	[7.5]	4.0	[6.2]

T0-T13 reflect time points in blocks of four weeks, where T0 is a pre-intervention baseline period and T1-13 the intervention period. [a]n=138 missing values; [b]n=7 missing values; [c]n=10 missing values; [d]n=15 missing values; [e]n=29 missing values; [f]n=30 missing values; [g]n=37 missing values; [h]n=56 missing values; [i]n=99 missing values; [j]n=98 missing values; [k]n=101 missing values; [l]n=103 missing values; [m]n=95 missing values; [n]n=107 missing values.

Table S9. Average (mean (SD)) pre-intervention and post-intervention sales per week for all intervention and all control supermarkets for each supermarket-level outcome (n=12).

	Control supermarkets		Intervention supermarkets	
	Pre-intervention sales	Post-intervention sales	Pre-intervention sales	Post-intervention sales
Total percentage of healthy product sales	27.4 (2.5)	27.6 (2.6)	27.5 (2.1)	27.5 (2.0)
Percentage sales within various food groups:				
Healthy fruits, vegetables, legumes and nuts sales	82.1 (3.1)	82.3 (2.6)	82.7 (3.9)	82.4 (3.2)
Healthy grain product sales	20.6 (3.2)	20.3 (3.3)	20.9 (2.6)	21.1 (2.8)
Healthy milk and yogurt products sales	35.6 (4.3)	36.0 (4.6)	36.2 (4.9)	35.9 (5.1)
Healthy cheese sales	16.9 (4.2)	18.2 (4.3)	18.2 (4.2)	19.9 (4.4)
Healthy meat products, meat substitutes and egg sales	18.4 (3.6)	14.4 (3.6)	20.2 (3.4)	15.5 (3.7)
Healthy fish sales	90.9 (2.7)	94.1 (2.7)	90.9 (3.4)	93.9 (3.8)
Healthy oils, fats and herbs and spices sales	25.8 (4.1)	25.7 (3.9)	25.5 (4.4)	25.9 (4.4)
Healthy non-alcoholic beverage sales	20.7 (5.3)	21.4 (6.2)	21.8 (4.8)	21.3 (4.9)
Healthy product sales from remaining food products	14.4 (1.9)	14.5 (1.8)	13.7 (1.4)	13.7 (1.3)
Unhealthy sweet and savoury snack sales of total sales	12.5 (1.7)	12.1 (1.3)	12.7 (1.2)	11.8 (1.0)
Total sales revenue (Euros)	68062.6 (13823.4)	72699.9 (23419.3)	66498.5 (15623.2)	59064.5 (15942.4)
