# Supplementary material for: Real-world nudging, pricing, and mobile physical activity coaching was insufficient to improve lifestyle behaviours and cardiometabolic health: the Supreme Nudge parallel cluster-randomised controlled supermarket trial
Source: BMC Med. 2024 Feb 2;22:52. doi: 10.1186/s12916-024-03268-4 (PMC10835818; doi:10.1186/s12916-024-03268-4)
Supplement: Supplementary file 2 — Additional file 2: Figures S1-S5. Figures S1a-k—Photo examples of nudge intervention: Figure S2—Average diet quality scores over time for different age levels in the intervention group and the control group of the Supreme Nudge trial (n = 361); Figure S3—Average waist circumference among females over time for different age levels in the intervention group and the control group of the Supreme Nudge trial (n = 239); Figure S4—Average total cholesterol to HDL ratio over time for different age levels in the intervention group and the control group of the Supreme Nudge trial (n = 290); Figure S5—Mean implementation fidelity scores (range 1–5) per month over the intervention period of one year, by supermarket. [file 12916_2024_3268_MOESM2_ESM.pdf]

Real-world nudging, pricing and mobile physical activity coaching was insufficient to improve lifestyle behaviours and cardiometabolic health: the Supreme Nudge parallel cluster-randomised controlled supermarket trial

Supplementary figures

Josine M Stuber, et al

Figure S1a. Photo example nudge intervention: Healthy shelf layout (whole-grain products on eyelevel) ... 2

Figure S1b. Photo example nudge intervention: Healthy checkout ... 3

Figure S1c. Photo example nudge intervention: Healthy end of aisle ... 4

Figure S1d. Photo example nudge intervention: Healthy aisle basket ... 5

Figure S1e. Photo example nudge intervention: Shelf-labels ... 5

Figure S1f. Photo example nudge intervention: Shelf feedback strip ... 6

Figure S1g. Photo example nudge intervention: Shelf-stoppers ... 6

Figure S1h. Photo example nudge intervention: Healthy suggestions shelf-banner ... 7

Figure S1i. Photo example nudge intervention: Explanation of nudging symbols on shopping cart handle ... 8

Figure S1j. Photo example nudge intervention: Nudging symbols on shopping cart ... 9

Figure S1k. Photo example nudge intervention:Nudging symbols in shopping basket ... 9

Figure S2. Average diet quality (DHD15-index) scores over time for different age levels in the intervention group (nudging and pricing strategies) and the control group of the Supreme Nudge trial (n=361). Analysis was based on a linear mixed model including all follow-up time points and with random intercepts for supermarket location and for participant, including the diet quality baseline value as covariate. ... 10

Figure S3. Average waist circumference among females over time for different age levels in the intervention group (nudging and pricing strategies) and the control group of the Supreme Nudge trial (n=239). Analysis was based on a linear mixed model including all follow-up time points and with random intercepts for supermarket location and for participant, including the waist circumference baseline value as covariate. 11

Figure S4. Average total cholesterol to HDL ratio over time for different age levels in the intervention group (nudging and pricing strategies) and the control group of the Supreme Nudge trial (n=290). Analysis was based on a linear mixed model including all follow-up time points and with random intercepts for supermarket location and for participant, including the total cholesterol to HDL ratio baseline value as covariate. ... 12

Figure S5. Mean implementation fidelity scores (range 1-5) per month over the intervention period of one year, by supermarket. ... 13

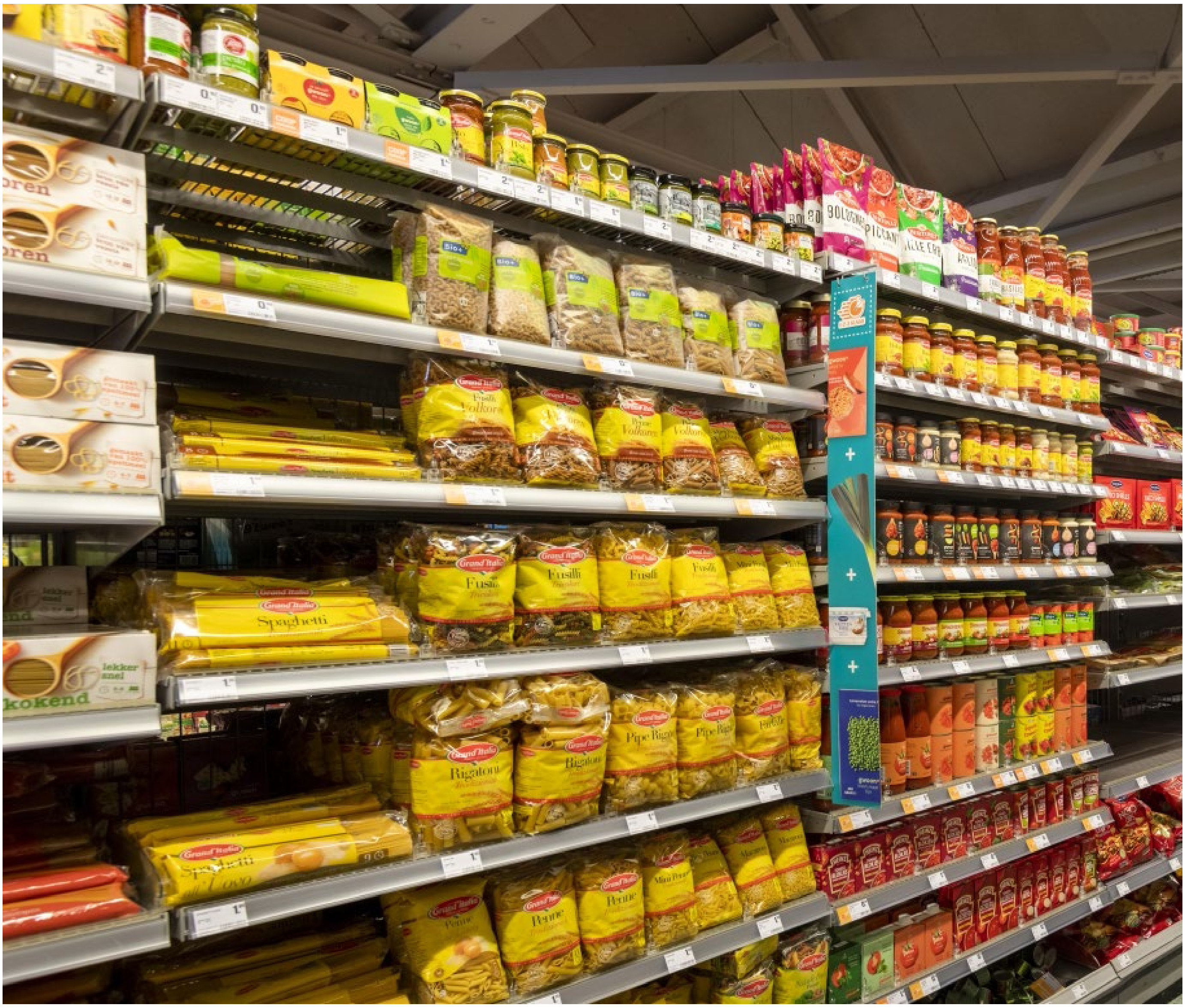

Figure S1a. Photo example nudge intervention: Healthy shelf layout (whole-grain products on eyelevel)

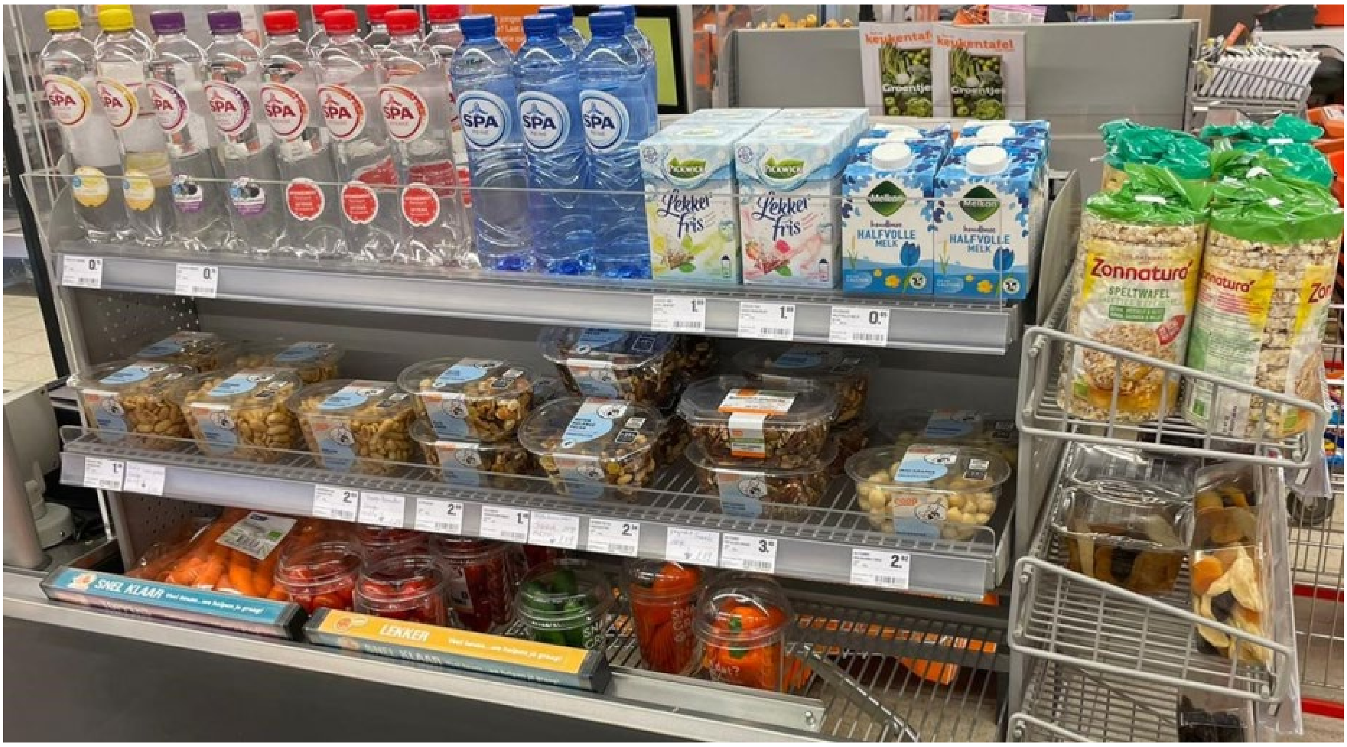

Figure S1b. Photo example nudge intervention: Healthy checkout

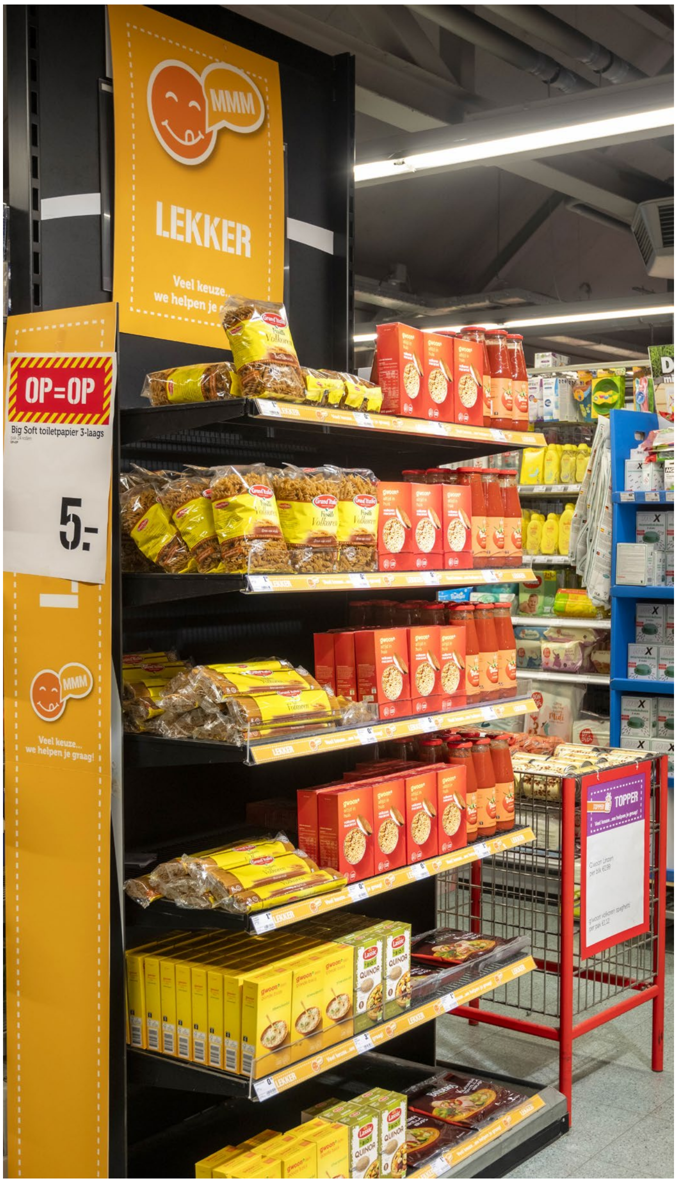

Figure S1c. Photo example nudge intervention: Healthy end of aisle

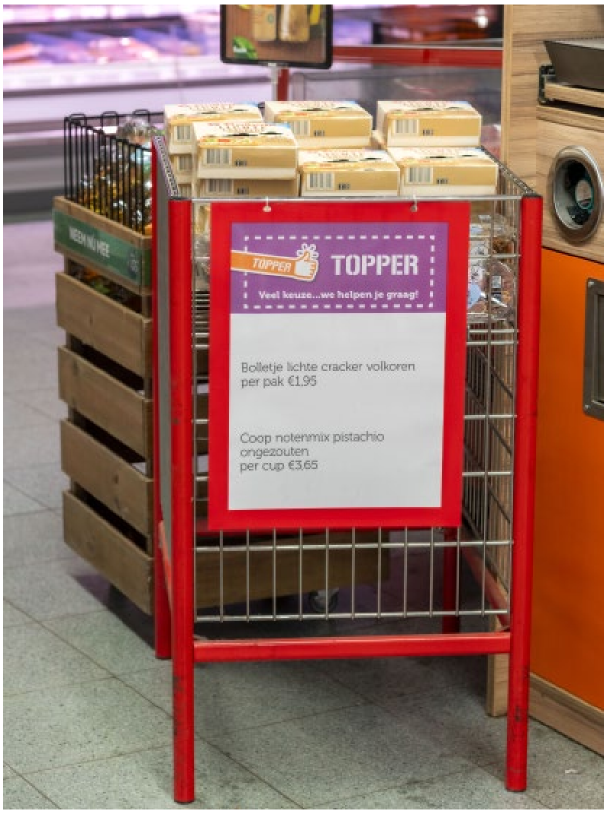

Figure S1d. Photo example nudge intervention: Healthy aisle basket

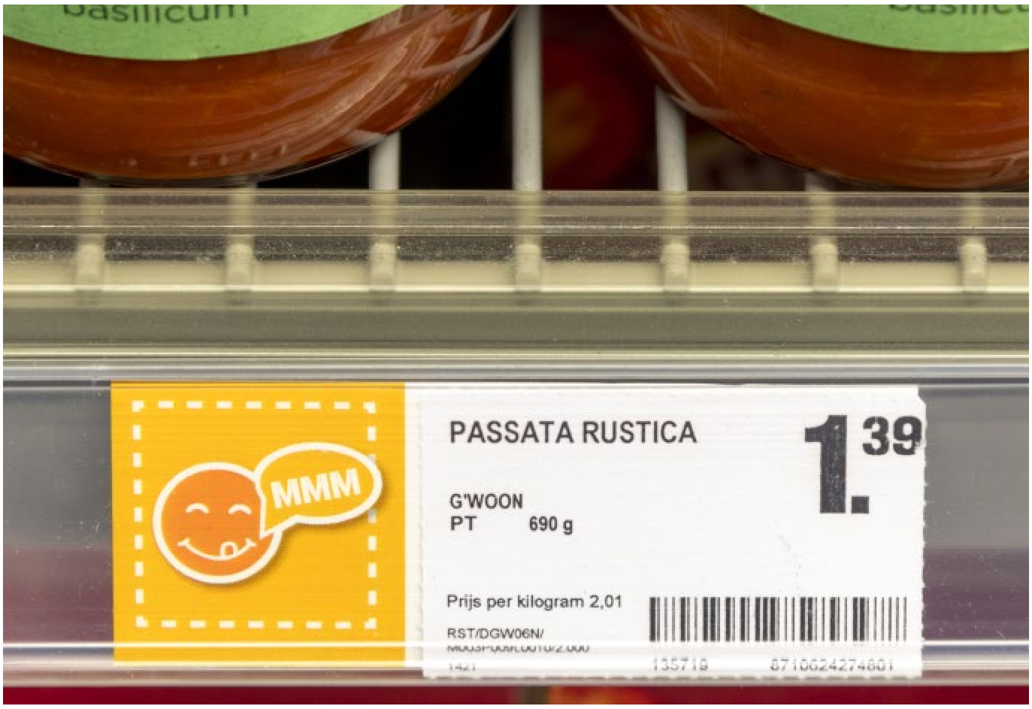

Figure S1e. Photo example nudge intervention: Shelf-labels

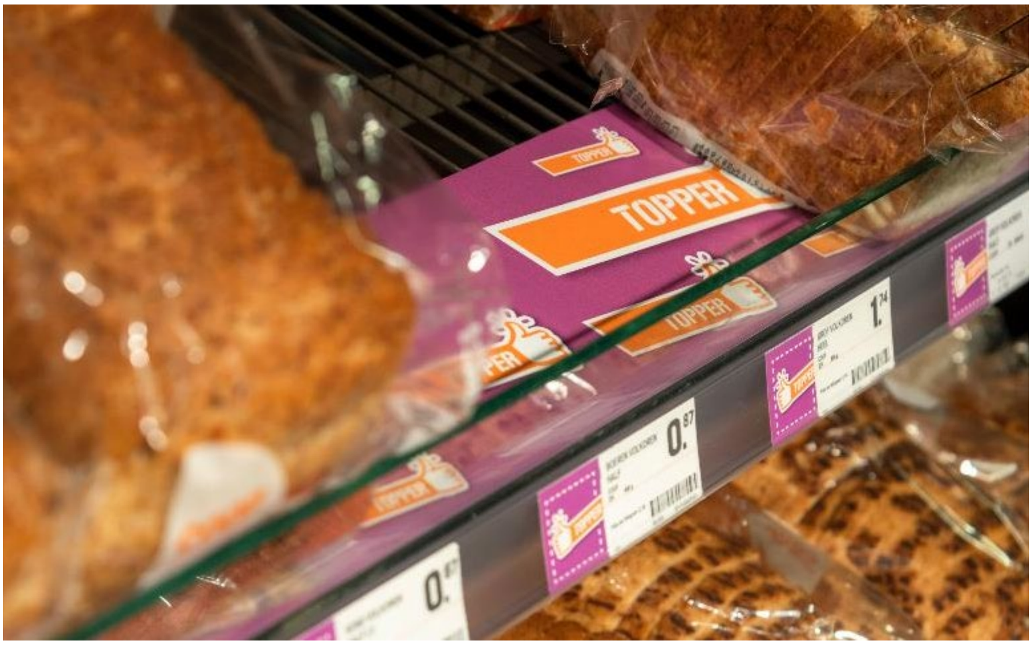

Figure S1f. Photo example nudge intervention: Shelf feedback strip

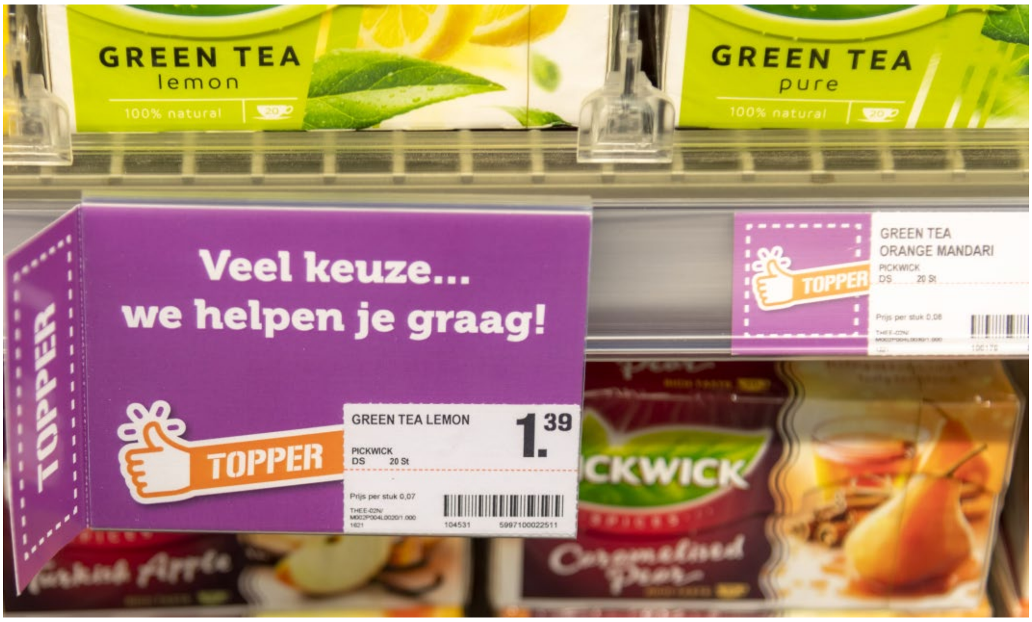

Figure S1g. Photo example nudge intervention: Shelf-stoppers

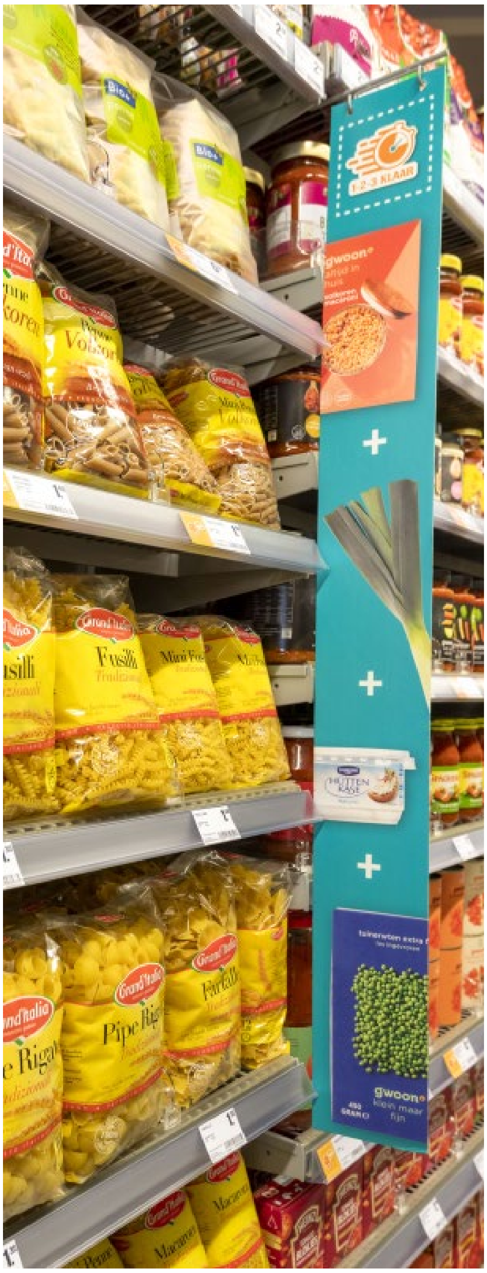

Figure S1h. Photo example nudge intervention: Healthy suggestions shelf-banner

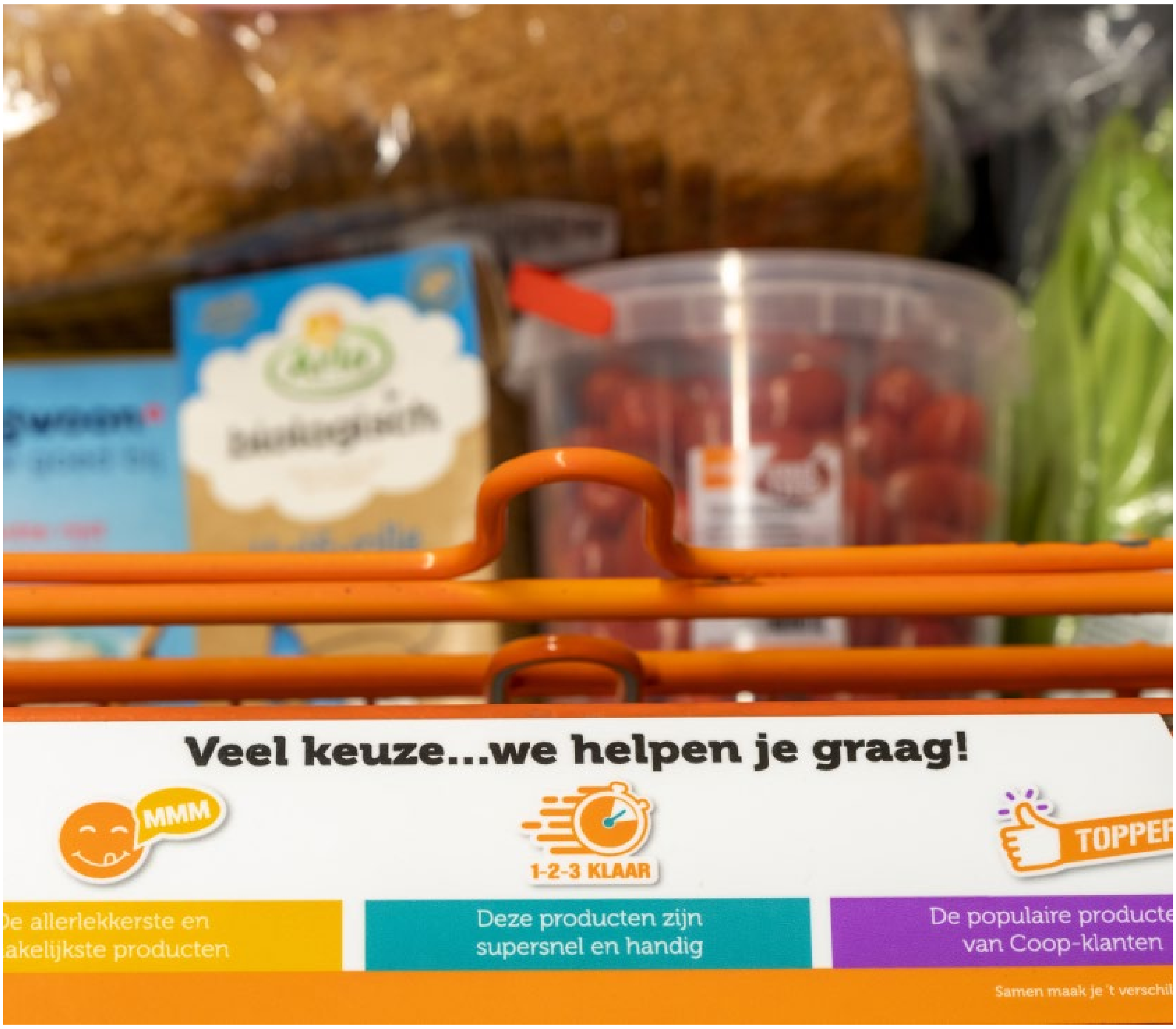

Figure S1i. Photo example nudge intervention: Explanation of nudging symbols on shopping cart handle

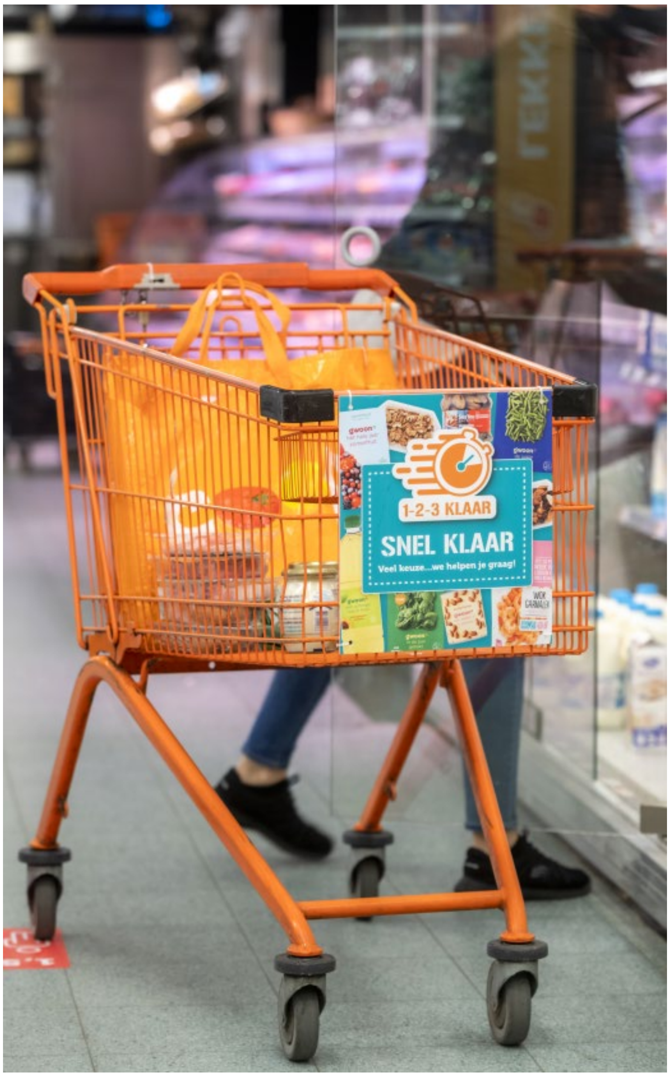

Figure S1j. Photo example nudge intervention: Nudging symbols on shopping cart

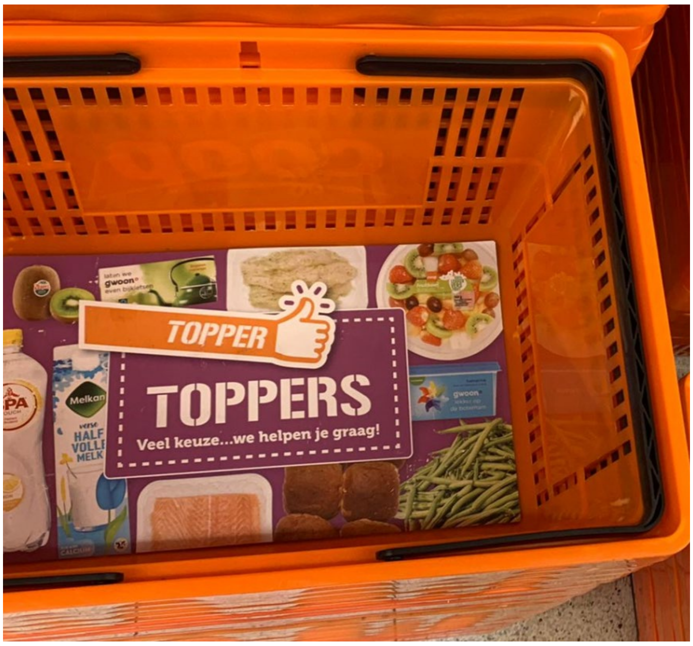

Figure S1k. Photo example nudge intervention:Nudging symbols in shopping basket

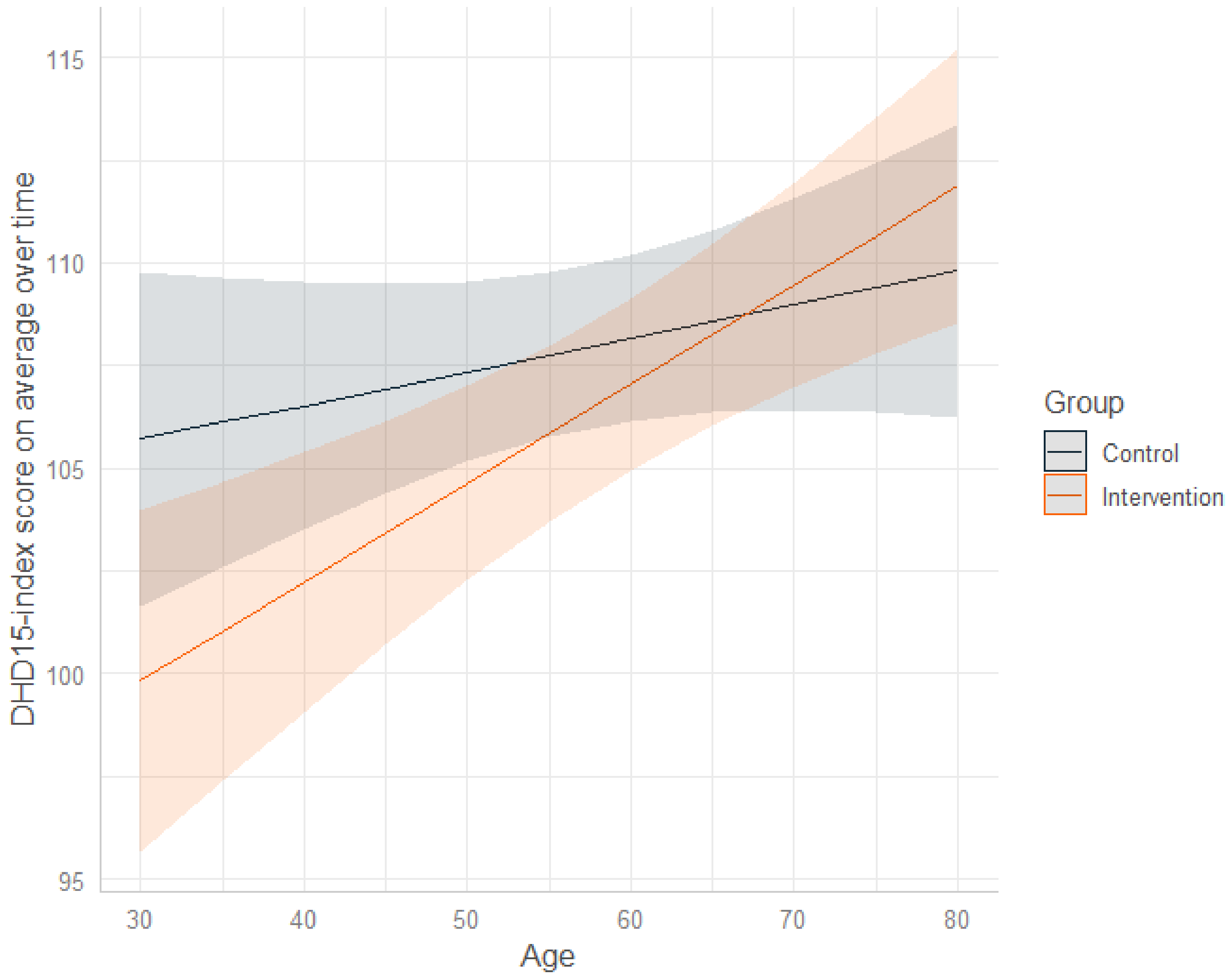

Figure S2. Average diet quality (DHD15-index) scores over time for different age levels in the intervention group (nudging and pricing strategies) and the control group of the Supreme Nudge trial (n=361). Analysis was based on a linear mixed model including all follow-up time points and with random intercepts for supermarket location and for participant, including the diet quality baseline value as covariate.

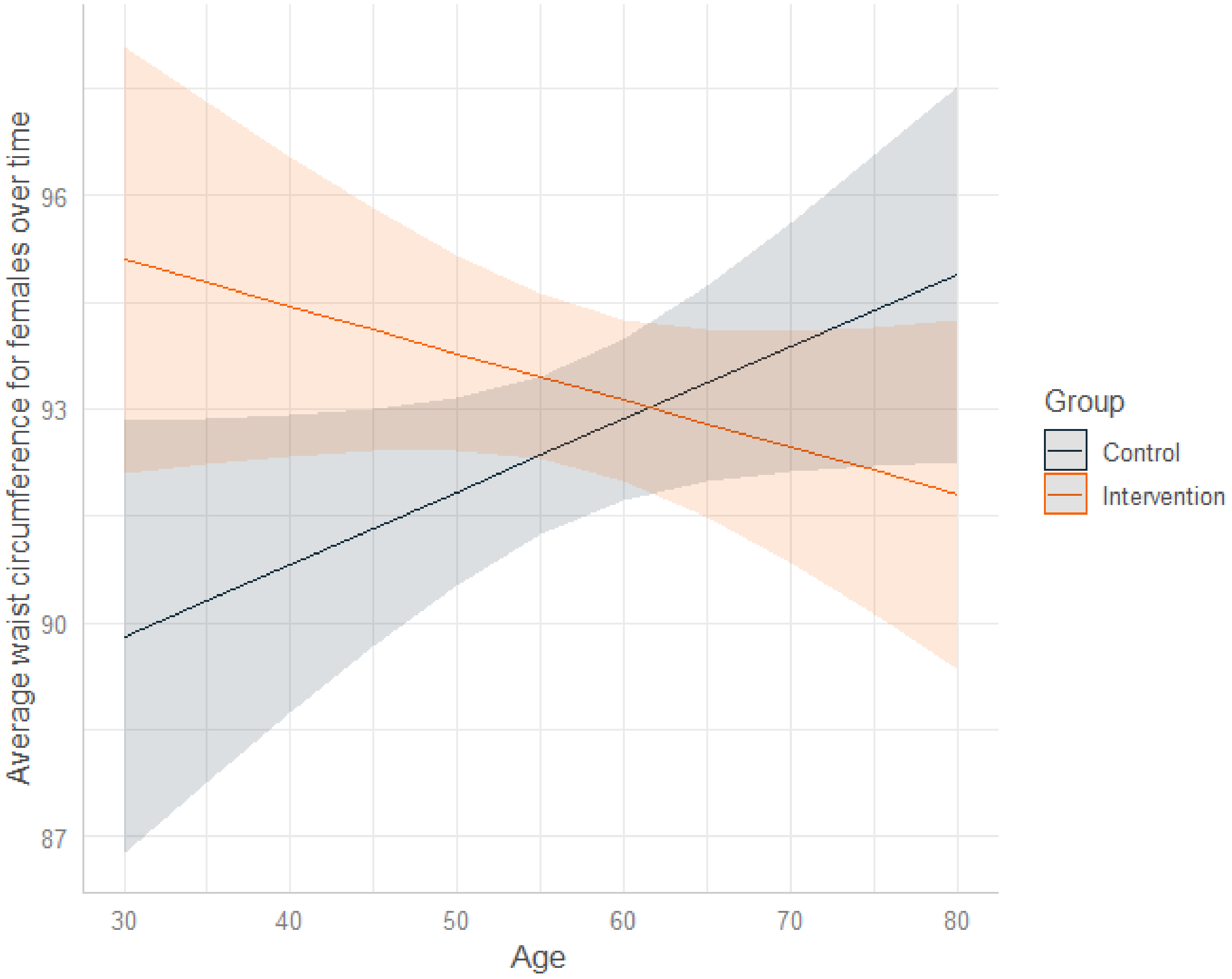

Figure S3. Average waist circumference among females over time for different age levels in the intervention group (nudging and pricing strategies) and the control group of the Supreme Nudge trial (n=239). Analysis was based on a linear mixed model including all follow-up time points and with random intercepts for supermarket location and for participant, including the waist circumference baseline value as covariate.

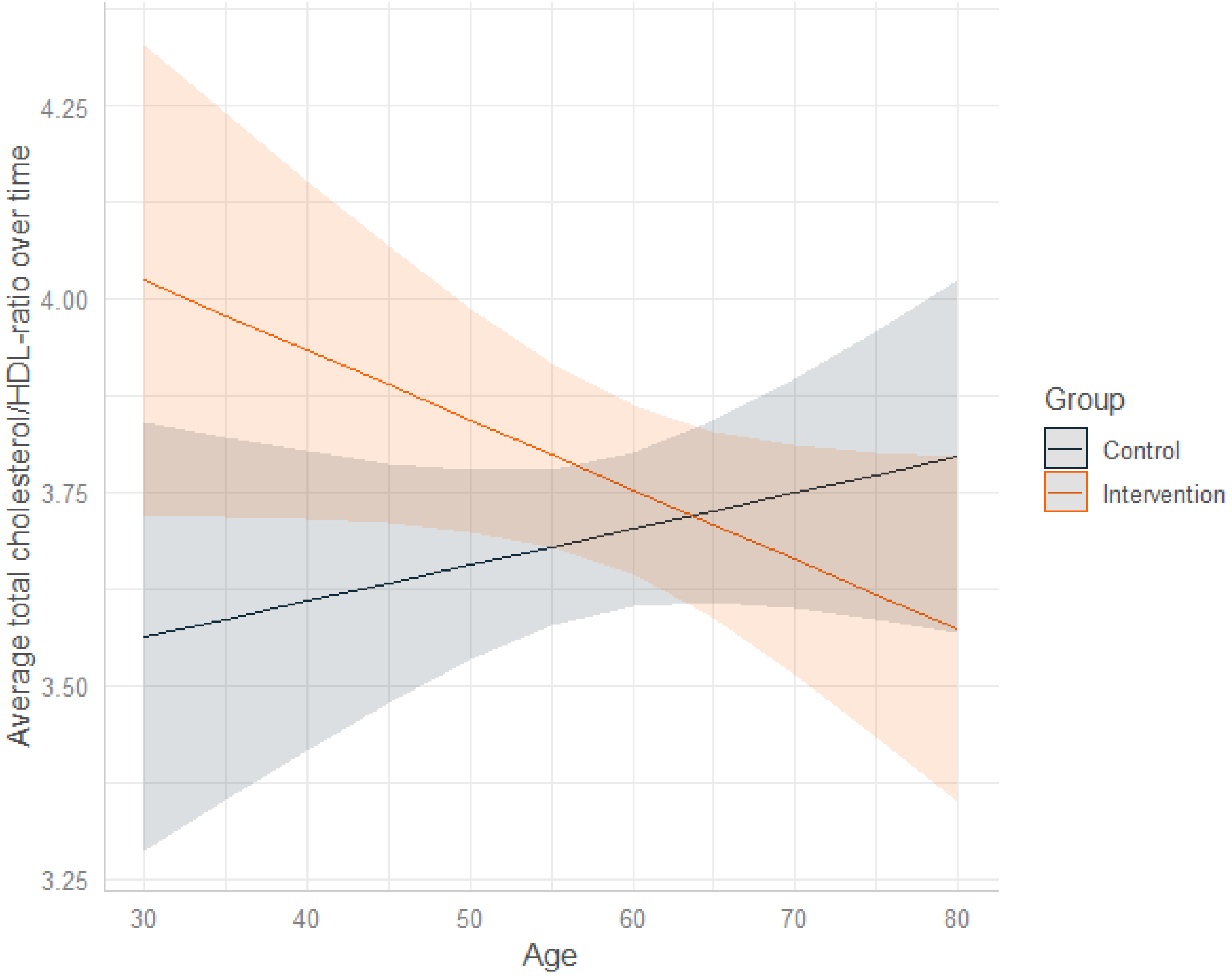

Figure S4. Average total cholesterol to HDL ratio over time for different age levels in the intervention group (nudging and pricing strategies) and the control group of the Supreme Nudge trial (n=290). Analysis was based on a linear mixed model including all follow-up time points and with random intercepts for supermarket location and for participant, including the total cholesterol to HDL ratio baseline value as covariate.

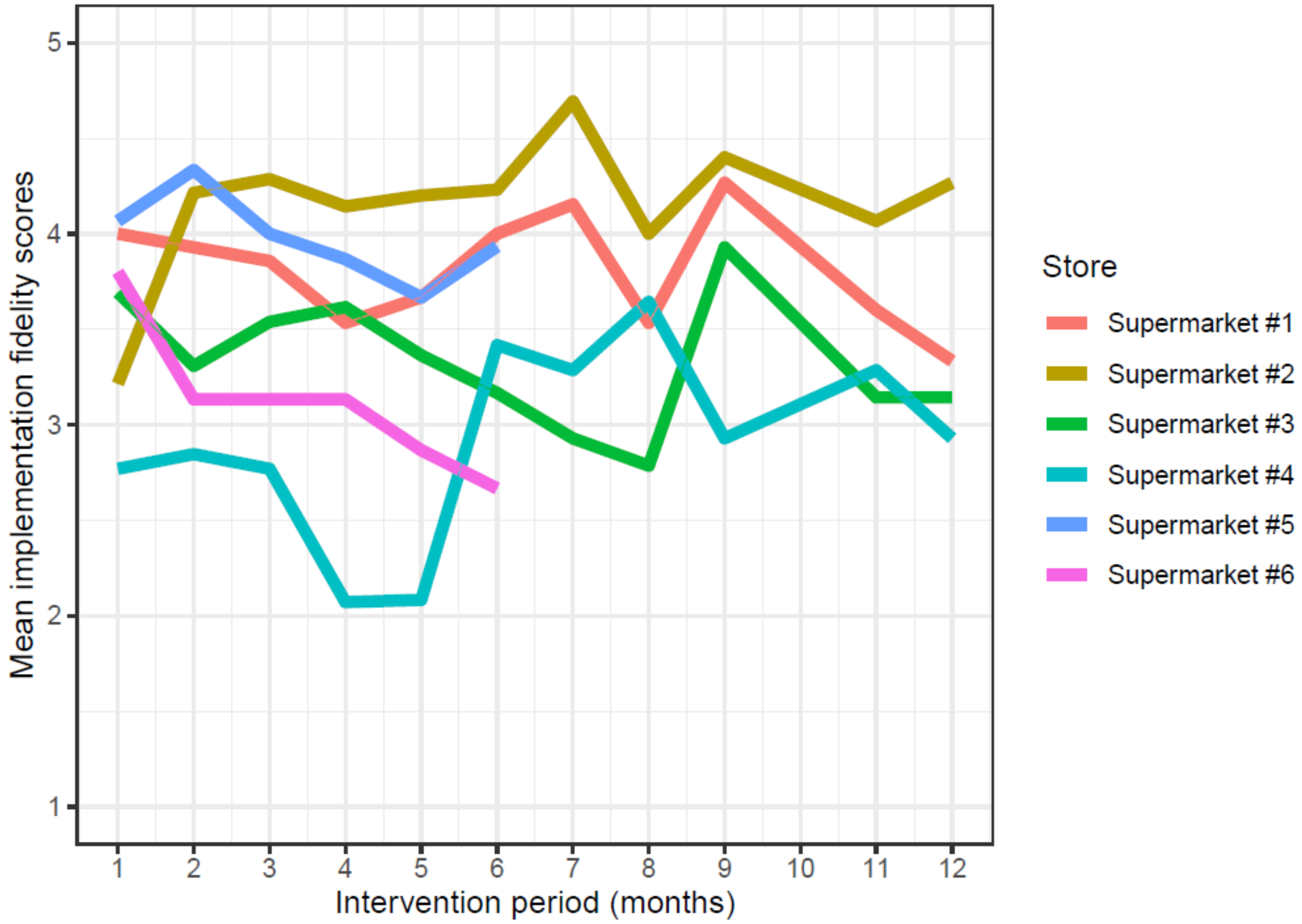

Figure S5. Mean implementation fidelity scores (range 1-5) per month over the intervention period of one year, by supermarket.
